# Divergent functions of hematopoietic transcription factors in lineage priming and differentiation during erythro-megakaryopoiesis

**DOI:** 10.1101/gr.164178.113

**Published:** 2014-12

**Authors:** Maxim Pimkin, Andrew V. Kossenkov, Tejaswini Mishra, Christapher S. Morrissey, Weisheng Wu, Cheryl A. Keller, Gerd A. Blobel, Dongwon Lee, Michael A. Beer, Ross C. Hardison, Mitchell J. Weiss

**Affiliations:** 1 Division of Hematology, The Children’s Hospital of Philadelphia, Philadelphia, Pennsylvania 19104, USA;; 2 Pediatric Residency Program, Department of Pediatrics, Children’s Hospital of Pittsburgh of UPMC, University of Pittsburgh School of Medicine, Pittsburgh, Pennsylvania 15224, USA;; 3 Center for Systems and Computational Biology, The Wistar Institute, Philadelphia 19019, Pennsylvania, USA;; 4 Center for Comparative Genomics and Bioinformatics, Pennsylvania State University, University Park, Pennsylvania 16802, USA;; 5 Department of Biochemistry and Molecular Biology, Pennsylvania State University, University Park, Pennsylvania 16802, USA;; 6 University of Pennsylvania Perelman School of Medicine, Philadelphia, Pennsylvania 19104, USA;; 7 McKusick-Nathans Institute of Genetic Medicine and Department of Biomedical Engineering, Johns Hopkins University School of Medicine, Baltimore, Maryland 21205, USA

## Abstract

Combinatorial actions of relatively few transcription factors control hematopoietic differentiation. To investigate this process in erythro-megakaryopoiesis, we correlated the genome-wide chromatin occupancy signatures of four master hematopoietic transcription factors (GATA1, GATA2, TAL1, and FLI1) and three diagnostic histone modification marks with the gene expression changes that occur during development of primary cultured megakaryocytes (MEG) and primary erythroblasts (ERY) from murine fetal liver hematopoietic stem/progenitor cells. We identified a robust, genome-wide mechanism of MEG-specific lineage priming by a previously described stem/progenitor cell-expressed transcription factor heptad (GATA2, LYL1, TAL1, FLI1, ERG, RUNX1, LMO2) binding to MEG-associated *cis*-regulatory modules (CRMs) in multipotential progenitors. This is followed by genome-wide GATA factor switching that mediates further induction of MEG-specific genes following lineage commitment. Interaction between GATA and ETS factors appears to be a key determinant of these processes. In contrast, ERY-specific lineage priming is biased toward GATA2-independent mechanisms. In addition to its role in MEG lineage priming, GATA2 plays an extensive role in late megakaryopoiesis as a transcriptional repressor at loci defined by a specific DNA signature. Our findings reveal important new insights into how ERY and MEG lineages arise from a common bipotential progenitor via overlapping and divergent functions of shared hematopoietic transcription factors.

Hematopoiesis is driven by networks of transcription factors that establish tissue-specific programs of gene expression (Orkin and Zon 2008; Kerenyi and Orkin 2010; Novershtern et al. 2011). Low-level expression of lineage-specific genes precedes lineage commitment in hematopoietic stem and progenitor cells (HSPCs), a process referred to as “lineage priming” (Hu et al. 1997; Miyamoto et al. 2002; Ng et al. 2009; Zandi et al. 2010; Novershtern et al. 2011). This correlates with the presence of specific “bivalent” chromatin states, although the underlying transcriptional mechanisms are unclear (Cui et al. 2009; Weishaupt et al. 2010; Zhou et al. 2011).

Erythrocytes and platelets originate from a common bipotential megakaryocyte-erythroid progenitor (MEP). A few lineage-restricted transcription factors, including erythroid (ERY)-enriched KLF1 (also known as EKLF) and megakaryocyte (MEG)-enriched FLI1 and ETS1, regulate the divergence of MEG and ERY phenotypes (Lulli et al. 2006; Orkin and Zon 2008; Kerenyi and Orkin 2010; Tallack et al. 2010; Novershtern et al. 2011). However, most transcription factors known to regulate erythro-megakaryopoiesis, including GATA1, GATA2, GFI1b, TAL1 (also called SCL), and FOG1, are shared between the two lineages with unique, essential roles in each (Hu et al. 1997; Miyamoto et al. 2002; Ng et al. 2009; Kerenyi and Orkin 2010; Zandi et al. 2010; Novershtern et al. 2011). Combinatorial interactions between transcription factors mediate cell type-specific chromatin binding and gene expression (Cheng et al. 2009; Cui et al. 2009; Fujiwara et al. 2009; Yu et al. 2009; Kassouf et al. 2010; Weishaupt et al. 2010; Wilson et al. 2010; Tijssen et al. 2011; Wu et al. 2011; Zhou et al. 2011; Dore et al. 2012). However, the molecular mechanisms of this selectivity are incompletely solved.

GATA1 and GATA2 are related transcription factors that recognize similar DNA motifs and act sequentially in blood development by orchestrating broad programs of gene activation and repression. Expression of GATA2 predominates in early hematopoiesis and declines during differentiation and maturation of most lineages, with a concomitant increase in GATA1 (Leonard et al. 1993; Grass et al. 2003). GATA1 gradually replaces GATA2 at a subset of its chromatin occupancy sites (OSs) in a process referred to as a “GATA switch” (Weiss et al. 1994; Bresnick et al. 2010; Kaneko et al. 2010; Snow et al. 2011; Dore et al. 2012). Genome-wide GATA switching at lineage-specific loci suggests a possible mechanism of hematopoietic lineage priming whereby GATA2 expressed in progenitor cells acts as a prebound factor prior to lineage-specific GATA1-mediated induction. However, although GATA1 completely replaces GATA2 in late erythropoiesis, substantial levels of GATA2 persist in MEG, suggesting distinct functions for the coexpressed GATA proteins (Visvader and Adams 1993; Chou et al. 2009).

This study examined the following unresolved questions related to the actions of GATA1, GATA2, and associated transcription factors in establishing and maintaining the ERY and MEG lineages: (1) What determines selective binding of GATA1 to lineage-specific *cis*-regulatory modules (CRMs)? (2) What is the scope and functional significance of GATA switching genome-wide, particularly in MEG? (3) How do the functions of GATA1 versus GATA2 overlap and differ in MEG? (4) To what extent does GATA switching play a role in hematopoietic lineage priming? We addressed these questions in primary cells (ERY) or primary cultured cells (MEG) expressing physiological levels of relevant transcription factors. As part of the Mouse ENCODE Project (The Mouse ENCODE Consortium et al. 2012; The Mouse ENCODE Consortium et al. 2014), we mapped the genome-wide chromatin occupancy of four master hematopoietic transcription factors (GATA1, GATA2, TAL1, and FLI1) and three histone methylation marks (H3K4me1, H3K4me3, and H3K27me3) in lineage-committed ERY and MEG cells. In addition, we defined global gene expression changes that accompany the development of these mature lineages from HSPCs. By correlating gene expression with transcription factor occupancy, we produce a global functional annotation of chromatin dynamics during erythro-megakaryopoiesis. Our findings provide new insights into GATA protein functions and reveal a robust, genome-wide mechanism of MEG lineage priming in multipotential hematopoietic progenitors.

## Results

### GATA1 and TAL1 regulate broad, divergent transcriptional programs in erythro-megakaryopoiesis

**Figure 1.**
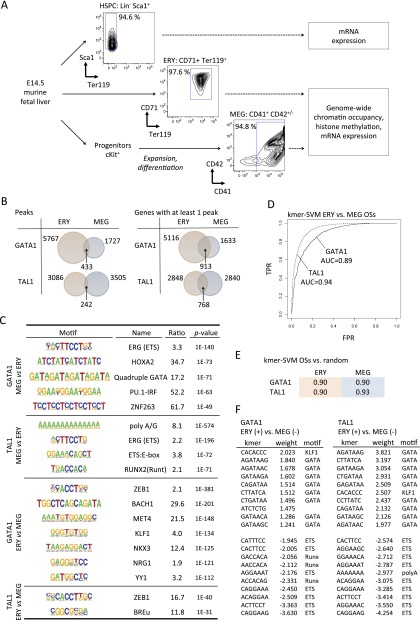
Context-specific functions for GATA1 and TAL1 in erythro-megakaryopoiesis. (*A*) Experimental scheme for deriving and analyzing hematopoietic stem cell and progenitor cells (HSPCs), erythroblasts (ERY), and megakaryocytes (MEG) from murine fetal liver. (*B*) Venn diagrams showing the intersection between ERY and MEG of transcription factor occupancy site (OS) peaks (Peaks) and transcription factor-bound genes (Genes). The latter is defined as a gene with at least one OS peak mapping between 10 kb upstream of the TSS and 3 kb downstream from the polyadenylation signal (Cheng et al. 2009; Wu et al. 2011). Genes containing multiple OSs were counted only once. (*C*) Motifs overrepresented in the 200-bp sequences surrounding the GATA1 peak center in ERY versus MEG, and vice versa. (*D*) ROC curves for kmer-SVM on GATA1 OSs in ERY versus MEG and TAL1 OSs in ERY versus MEG. (TPR) True positive rate; (FPR) false positive rate. (*E*) kmer-SVM results (AUC) for GATA1 and TAL1 OSs trained against random sequences. (*F*) High weight *k*-mers correspond to known cofactor binding sites. In ERY, KLF1 motifs predict GATA1 binding and GATA motifs predict TAL1 binding. In MEG, ETS and RUNX motifs are positive predictors of GATA1 and/or TAL1 binding.

We generated and compared the following murine hematopoietic populations (Fig. 1A): primary fetal liver-derived erythroblasts (Ter119^+^ CD71^high^; >97% purity); megakaryocytes cultured from primary fetal liver HSPCs (CD41^+^ CD42^+^; >94% purity), and HSPCs (lin^−^ Sca1^+^ Ter119^−^; >94% purity). (Details about the cell purification and culture are given in the Methods, Supplemental Methods, and Supplemental Fig. 1.) We mapped GATA1 and TAL1 occupancy sites (OSs) in fetal liver-derived MEG and ERY cells by chromatin immunoprecipitation followed by massively parallel DNA sequencing of transcription factor-bound DNA (ChIP-seq) (Fig. 1A). We used irreproducible discovery rate at a threshold of 0.02 to determine the number *n* of reproducible peaks in our replicate data sets as a high-stringency, conservative peak calling approach (Li et al. 2011; Landt et al. 2012). For cases in which the smaller number of peaks identified by the approach could bias data interpretation, findings were confirmed using a larger set of reduced stringency peaks (Supplemental Methods).

The genome-wide occupancy patterns were overwhelmingly lineage-specific (Fig. 1B; Supplemental Figs. 2, 3) with only a minority of GATA1 and TAL1 OSs overlapping between ERY and MEG. The distribution of GATA1 and TAL1 OSs across the fetal liver erythroblast genome was similar to what we observed in the G1E erythroid cell line (Cheng et al. 2009). Specifically, most OSs occurred near or within a gene neighborhood, defined as a region spanning 10 kb upstream of and 3 kb downstream from the TSS (Supplemental Fig. 3). The majority of OSs occurred within introns, especially the first intron (Supplemental Figs. 4, 5), although there was a distinct peak of transcription factor density at transcription start sites (TSSs) (Supplemental Fig. 6). Some genes were occupied by TAL1 and/or GATA1 in both lineages, but in most of these cases, transcription factor binding tended to occur at geographically distinct sites, indicating widespread utilization of lineage-specific CRMs in individual gene loci (Fig. 1B and Supplemental Fig. 2).

### Distinct combinations of transcription factor binding motifs contribute to lineage-specific chromatin occupancy

MEG-specific GATA1 and GATA2 OSs are enriched for ETS factor binding motifs (Deveaux et al. 1996; Holmes et al. 2002; Wang et al. 2002; Hazony et al. 2006; Pang et al. 2006; Dore et al. 2012). Having occupancy data for the same transcription factors in two different cell lineages allowed us to search for informative motifs that were not revealed by earlier enrichment analyses of single data sets. We applied the HOMER algorithm for de novo motif discovery, searching for motifs enriched in the OSs from one lineage (e.g., MEG) that are not enriched in the alternate lineage (e.g., ERY) (Heinz et al. 2010). In addition to the enrichment of ETS motifs in GATA1 and TAL1 OS specifically in MEG, we also detected enrichment of several additional DNA motifs, some of which have not been previously implicated in hematopoiesis (Fig. 1C). This discriminative approach further refines the complex DNA signatures of ERY and MEG-specific CRMs.

To define a combination of DNA motifs that is sufficient to predict lineage specific binding, we used a complementary discriminator based on a support vector machine (SVM) framework, kmer-SVM (Lee et al. 2011). The SVM was trained to maximally separate DNA segments bound by GATA1 in MEG versus those bound in ERY based on sequence features. Specifically, we examined the number of occurrences of short nucleotide strings of length *k* (*k*-mers, *k* ranging from 3 to 10). We used the kmer-SVM to discriminate between GATA1 OSs in ERY and MEG, and between TAL1 OSs in ERY and MEG. In each case the program was able to classify the test set sequences with high accuracy, both when OS sets were trained against each other (Fig. 1D) and against a random background (Fig. 1E). The largest weight (i.e., most significant) cofactor *k*-mers were for ETS and RUNX in MEG and KLF1 in ERY (Fig. 1F), but *k*-mers corresponding to binding sites for other cofactors including ZEB1, AP1, and novel elements were also detected (Fig. 1F and data not shown). Although confirming the enrichment analysis, the independent kmer-SVM results also show that these combinations of sequence motifs are sufficient to accurately distinguish MEG versus ERY specific binding at sites bound by GATA1 and TAL1 throughout the genome.

### GATA1-TAL1 and GATA1-FLI1 complexes activate MEG genes

**Figure 2.**
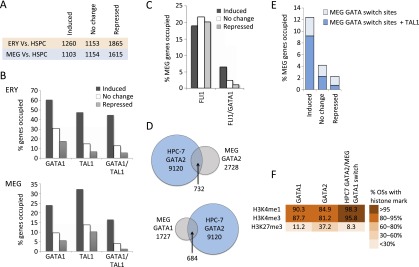
Functional annotation of GATA1, GATA2, and FLI1 occupancy in megakaryopoiesis. (*A*) Numbers of up-regulated (induced), unchanged, and down-regulated (repressed) genes in ERY and MEG compared to HSPCs, used as a reference point. (*B*) Fractions of up-regulated, unchanged, and down-regulated genes (versus HSPC) occupied by GATA1 or TAL1 (irrespective of co-occupancy by other transcription factors) and genes co-occupied by GATA1 and TAL1 in ERY and MEG. (*C*) Fraction of up-regulated, unchanged, and down-regulated genes occupied by FLI1 in MEG (all FLI1 OSs and those co-occupied by GATA1 and FLI1). (*D*) Intersection of transcription factor occupancy peaks between MEG and the multipotent hematopoietic cell line HPC-7 (Wilson et al. 2010). (*E*) Fractions of up-regulated, unchanged, and down-regulated genes containing a GATA switch site (replacement of GATA2 with GATA1) in MEG development, with or without concurrent TAL1 binding. (*F*) Color-coded fractions of transcription factor binding sites associated with H3K4me3, H3K4me1, and H3K27me3 histone methylation patterns in MEG.

GATA factor functions are influenced by protein interactions (Kim and Bresnick 2007; Bresnick et al. 2010; Kaneko et al. 2010). For example, association of GATA1 with TAL1 stimulates gene activation in erythropoiesis (Cheng et al. 2009; Tripic et al. 2009; Yu et al. 2009). In MEG, gene activation is augmented by GATA1 synergy with ETS family factors FLI1 and GABPA (Wang et al. 2002; Pang et al. 2006). We set out to correlate GATA1 and TAL1 genome occupancy patterns with mRNA expression in ERY and MEG lineages. We interrogated a HSPC population (fetal liver Sca1^+^, lin^–^), MEG and ERY transcriptomes on cDNA microarrays (Supplemental Fig. 7), and used the HSPC transcriptome as a reference point to define the developmental changes in gene expression during monolineage differentiation. Of 28,853 genes interrogated, 7513 were significantly expressed in at least one population (Supplemental Methods). We designated genes as developmentally induced or repressed if their mRNA expression significantly (FDR < 5%) changed more than twofold relative to HSPC (Fig. 2A). Although some genes are known to be regulated by CRMs that are quite far from a promoter, our ability to discern targets of distal CRMs is in its infancy. Thus, we confined our correlative analysis to genes with transcription factor occupancy within their neighborhood (10 kb upstream of and 3 kb downstream from the TSS), making the simplifying assumption that each gene was regulated by transcription factors bound within this interval. In both lineages, co-occupancy by both GATA1 and TAL1 was associated with increased likelihood of gene induction (Fig. 2B). Although this correlation is common to both cell types, most of the induced genes differ in the two lineages.

We also mapped the genomic locations occupied by the ETS family member FLI1 in MEG. FLI1 occupancy correlated roughly equally with activation, maintenance, or inhibition of gene expression (Fig. 2C) and showed a strong predilection for the TSS region, where its binding most frequently associated with gene repression (Supplemental Fig. 8A,B). However, FLI1 colocalized with GATA1 at a minority (15%) of the FLI1 OSs, and this association strongly correlated with gene induction (Fig. 2C). Thus, the previously described model of megakaryocyte-specific gene activation by GATA1/FLI1 co-occupancy (Wang et al. 2002) occurs genome-wide, involving at least 72 MEG-induced genes (data not shown).

### Distinct genome-wide GATA switches in MEG and ERY development

During ERY and MEG maturation, GATA2 and GATA1 undergo chromatin occupancy switches, in which GATA1 replaces HSPC-expressed GATA2 at a subset of GATA OSs (Kaneko et al. 2010; Dore et al. 2012). In order to obtain a direct measure of GATA switching during hematopoiesis, we compared our chromatin occupancy data with the previously published genome-wide occupancy map of GATA2 in murine HSPC. Since it is not yet feasible to obtain sufficient quantities of primary HSPCs for genome-wide ChIP-seq to analyze chromatin-bound transcription factors, we examined data generated from the multipotent line HPC-7 (Wilson et al. 2010), which is capable of MEG, ERY, and myeloid differentiation (Pinto do Ó et al. 1998). Up to 40% of MEG GATA1 OSs and 27% of MEG GATA2 OSs overlapped with GATA2 occupancy in HPC-7 cells (Fig. 2D). This indicates that extensive GATA factor binding in MEG is established in HSPCs and provides evidence for a genome-wide GATA switch in MEG development. In contrast, only 15% of ERY GATA1 OSs were occupied by GATA2 in HPC-7 (Supplemental Fig. 9), indicating that most GATA1 functions during erythropoiesis occur via de novo binding during cellular maturation rather than developmental transcription factor switching at CRMs prebound by GATA2. Notably, GATA switch sites in MEG were enriched for TAL1 and were strongly associated with gene induction (Fig. 2E). In MEG, these CRMs are associated with a high prevalence of activating H3K4me1 and H3K4me3 histone methylation marks and a comparatively low prevalence of the repressive H3K27me3 histone methylation, consistent with a transcription activating function (Fig. 2F).

### GATA-ETS factor elements are associated with gene silencing in ERY

**Figure 3.**
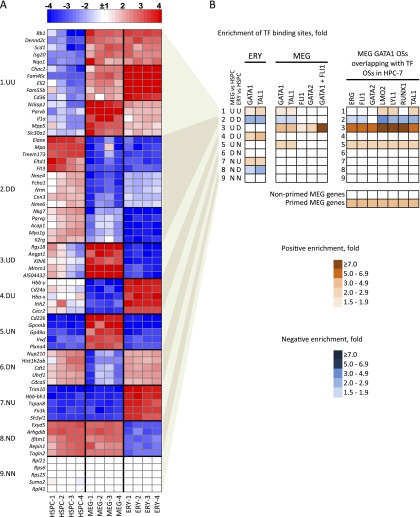
Mechanisms of transcriptional regulation correlate with patterns of erythro-megakaryocytic gene expression. (*A*) Heatmap of mRNA expression in HSPCs, MEG, and ERY with top changed genes clustered into nine groups according to patterns of expression relative to that of HSPCs. Data from four biological replicates are shown for each lineage. In each cluster, a nonbiased selection of top changed genes is shown: (U) up-regulated; (D) down-regulated; (N) no change. For example, cluster 3, labeled “UD,” represents genes that are up-regulated in MEG and down-regulated in ERY versus HSPCs. Total numbers of genes in each cluster, as well as gene function enrichments, are shown in Supplemental Figure 10A. (*B*) Over- or underrepresentation of transcription factor occupancy within ERY and MEG genes across the nine expression pattern clusters described in *A*. The enrichment value is a ratio of the fraction of genes in a given cluster occupied by a given transcription factor versus the fraction of occupied genes in the global expressed gene set of 7513 genes. The color-coding shows positive or negative enrichment of transcription factor binding in each gene cluster relative to the genome-wide average of binding probability (indicated by white color). All enrichments shown have *P-*values < 0.001 by Fisher’s exact test. The *right* panel shows enrichments of overlapping of MEG GATA1 OSs with indicated transcription factor OSs in HPC-7 hematopoietic progenitor cells (see Fig. 2; Wilson et al. 2010). The *bottom* panel shows enrichment of transcription factor binding in primed versus nonprimed MEG genes.

Transcriptome data demonstrate distinct patterns of gene expression in MEG versus ERY (Fig. 3A; Supplemental Fig. 7A). We further separated the 7513 HSPC+MEG+ERY expressed genes into nine clusters based on the combined directions of their expression changes compared to HSPCs (Fig. 3A; Supplemental Fig. 10A). We then quantified the enrichment of transcription factor binding across the nine clusters (Fig. 3B). Unexpectedly, GATA2, FLI1, and especially GATA1/FLI1-co-occupied MEG CRMs were selectively enriched in genes that were developmentally induced in MEG and repressed in ERY (Fig. 3B, cluster 3). This observation suggests that an interaction between GATA1 and FLI1 in early progenitors marks MEG-specific genes that are subsequently silenced upon commitment to the ERY lineage. Indeed, a DNA motif search comparing the sequences of GATA1-occupied CRMs in cluster 1 (genes induced in both MEG and ERY) versus cluster 3 demonstrated a significant enrichment of ETS motifs in the latter, which represents MEG-induced, discordantly regulated genes (Supplemental Fig. 10B). Thus, interaction between GATA1 and ETS factors appears to regulate selectively a distinct, MEG-specific set of genes that are expressed at a low level in HSPC, further induced during MEG differentiation and silenced in the ERY lineage.

### A stem/progenitor cell-expressed transcription factor heptad (GATA2, LYL1, TAL1, FLI1, ERG, RUNX1, LMO2) mediates MEG lineage priming

Our data point toward a genome-wide role for combinatorial actions of GATA and ETS factors in MEG lineage priming of genes that are eventually repressed upon ERY differentiation. To obtain direct evidence of transcription factor binding at these MEG specific loci in HSPCs, we again compared our chromatin occupancy map with those from HPC-7 cells (Wilson et al. 2010). Remarkably, >60% of MEG FLI1 OSs and >85% of MEG GATA1/FLI1 OSs were also occupied by FLI1 in HPC-7 cells (Supplemental Fig. 8D). Thus, in HSPC, FLI1 is prebound at most MEG-specific loci. FLI1 is one of a “heptad” of transcription factors (along with GATA2, LYL1, TAL1, ERG, RUNX1, and LMO2) noted to co-occupy specific DNA OSs in HPC-7 cells (Wilson et al. 2010). Although referred to as a heptad, this complex likely also contains LDB1, a protein that does not bind directly to DNA but mediates interactions among GATA factors, LMO2, and TAL1 (Kerenyi and Orkin 2010). These “heptad”-bound sites were highly and specifically enriched in the cluster of genes induced during MEG differentiation and repressed in ERY (Fig. 3B, cluster 3; Supplemental Fig. 11). The promoters of these genes are enriched for GATA, E-box, and ETS motifs (Supplemental Fig. 10A, cluster 3). Our findings indicate that overlapping sets of related transcription factors occupy the same MEG-specific gene regulatory elements continuously, beginning in HSPC populations and extending throughout MEG commitment and differentiation.

**Figure 4.**
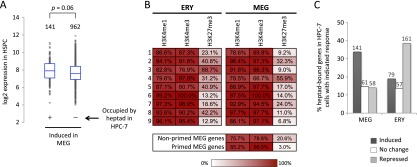
Characteristics of MEG lineage-primed genes. (*A*) Box-and-whisker plots of baseline expression levels in HSPCs of MEG-induced genes that are bound by the GATA2/LYL1/TAL1/FLI1/ERG/RUNX1/LMO2 transcription factor heptad in HPC-7 cells (Wilson et al. 2010) versus MEG-induced genes with no heptad occupancy in HPC-7 cells. (*B*) Color-coded distribution of histone methylation marks in the gene clusters defined in Figure 3A. Numbers indicate percentage of genes with H3K4me3, H3K4me1, and H3K27me3 methylation marks within 0.5 kb surrounding the TSS. (*C*) Fractions of GATA2/LYL1/TAL1/FLI1/ERG/RUNX1/LMO2 heptad-occupied genes in HPC-7 that are induced, unchanged, or repressed in MEG and ERY development, respectively. The numbers of genes in each group are indicated *above* the bars.

To correlate transcription factor binding with expression levels in HSPCs, we considered two groups of MEG-induced genes: (1) genes classified as nonexpressed in HSPC (mRNA levels less than 2× array background), and (2) genes with higher starting expression levels (greater than 10-fold array background). These categories represent more extreme cases of transcriptionally nonprimed (i.e., silent prior to induction) and primed (expressed in HSPC prior to full induction in MEG) genes, respectively. There is selective enrichment of overlapping MEG/HPC-7 OSs in the “primed” gene category (Fig. 3B, right). Indeed, binding of the heptad in HPC-7 is associated with higher starting expression levels in the primary HSPC population examined by us (Fig. 4A). Silencing of primed MEG-specific genes in ERY is associated with a loss of the activating H3K4me1 and H3K4me3 histone methylation marks and accumulation (or retention) of the repressive H3K27me3 histone modification (Fig. 4B, line 3). Thus, the heptad transcription factor complex occupies an extensive set of MEG-specific genes in HSPCs. Binding of this heptad is associated with low-level gene expression in primary HSPCs, and subsequent further induction in committed MEG, and is detected in 141 of 1103 MEG-induced genes (Fig. 4A; Supplemental Fig. 12). Many MEG-enriched genes, including *Itga2b* (CD41), *Ppbp* (platelet basic protein), *Pf4* (platelet factor 4), and *Gp1ba* (GP1B-alpha/CD42B alpha) appear to be regulated via this mechanism (Supplemental Table 1). These findings indicate the existence of a robust, genome-wide mechanism of MEG lineage priming. Given that our stringent criteria for peak calling and gene selection inevitably excluded many genes from analysis, we predict that the actual scope of MEG gene priming by the transcription factor heptad is substantially greater.

A smaller fraction of ERY-induced genes appears to use a similar priming mechanism using the same heptad (Supplemental Fig. 13), but most of these genes are also induced in the MEG and are therefore not ERY-specific (Fig. 3A,B, cluster 1; Supplemental Fig. 12B; Supplemental Table 2). Indeed, the divergent, lineage-specific functions of the transcription factor heptad are demonstrated by the fact that genes bound by the heptad in HPC-7 cells tend to be induced in MEG and repressed in ERY (Fig. 4C). Thus, in contrast to MEG gene expression, the transcriptional priming of most ERY-specific genes occurs primarily via GATA2-independent mechanisms.

### Examples of developmentally primed genes

**Figure 5.**
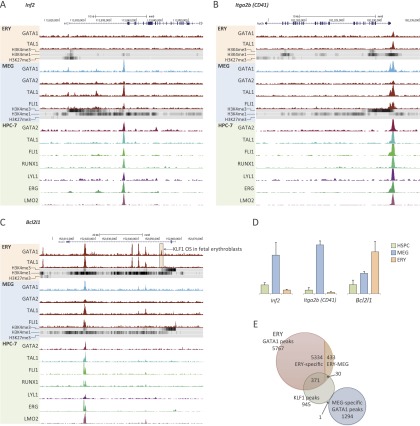
Developmentally regulated loci in erythro-megakaryopoiesis. (*A–C*) Selected genes are shown with the direction of transcription indicated by arrows and exons represented by black rectangles. ChIP-seq data showing transcription factor occupancy and histone marks in ERY, MEG, and HPC-7 cells are indicated *below* (Wilson et al. 2010). A GATA1 OS (*boxed*) in the *Bcl2l1* gene (*C*) overlaps with a KLF1 OS identified previously in murine fetal liver erythroblasts (Tallack et al. 2010). (*D*) Graphs showing relative expression of *Inf2*, *Itga2b*, and *Bcl2l1* in HSPCs, MEG, and ERY. (*E*) Genome-wide intersection of GATA1 OSs from our study with a published genome-wide map of KLF1 OSs in E14.5 mouse fetal liver erythroblasts (Tallack et al. 2010).

A typical developmentally primed gene, *Inf2* (Fig. 5A) coding for inverted formin-2, is expressed at low level in HSPCs and induced fourfold in MEG (Fig. 5D). In HPC-7 cells, it is occupied by the GATA2/FLI1/LYL1/TAL/ERG/RUNX1/LMO2 heptad at a +10.5 enhancer in intron 1. In MEG, the enhancer remains occupied by FLI1 and TAL1, but undergoes a GATA switch in which GATA2 is replaced with GATA1. The enhancer contains juxtaposed GATA, ETS, and TAL1 (E-box) factor motifs (data not shown). In ERY, where *Inf2* is silenced, the MEG-specific enhancer is not occupied by GATA1 or TAL1, and its epigenetic signature includes decreased H3K4me1 and H3K4me3, whereas the promoter acquires (or retains) the repressive H3K27me3 methylation mark. Many MEG-specific genes that mediate platelet functions, including selectin, platelet (*Selp*), platelet factor 4 (*Pf4*), glycoprotein 9 (platelet) (*Gp9*), and CD42B alpha (*Gp1ba*) are regulated in a similar fashion (not shown).

*Itga2b* (CD41) exemplifies a less typical MEG-primed gene (Fig. 5B). Like *Inf2*, it is primed in HSPC by the transcription factor heptad binding, in this case at a −0.3 element, which undergoes a GATA2 to GATA1 switch in late MEG development. Additional binding of GATA1, TAL1, and FLI1 also occur in MEG at an adjacent site. However, GATA1 and TAL1 remain bound at the original site in ERY, despite the gene being strongly repressed in this lineage (Fig. 5). Thus, *Itga2b* belongs to a limited set of MEG-specific genes in which GATA1, TAL1, or both remain bound in ERY but in a distinctive arrangement that is associated with gene silencing. This process may involve recruitment of additional lineage-specific repressors (see Fig. 7, discussed below). A full interpretation of such loci warrants direct functional studies of individual CRMs.

*Bcl2l1* exemplifies a developmentally primed gene that is induced during both ERY and MEG differentiation (Fig. 5C,D). The transcription factor heptad binds *Bcl2l1* at two closely spaced CRMs at +41 and +41.5, with additional low-level occupancy at +14. In MEG, GATA1, TAL1, and FLI1 target the same enhancers, while there are at least three additional, ERY-specific CRMs. *Bcl2l1* undergoes a greater degree of induction in ERY compared with MEG (Fig. 5D), and it is possible that one or more of the ERY-specific GATA1 OSs (Fig. 5D) contribute to this enhanced expression. At least one of these OSs is bound by KLF1 in mouse fetal erythroblasts (Fig. 5C, boxed; Tallack et al. 2010). Indeed, a substantial proportion of ERY-specific GATA1 OSs is also bound by KLF1 in fetal liver erythroblasts (Fig. 5E). This is consistent with KLF1 motif enrichment, specifically at ERY GATA1 OSs (Fig. 1C,F), and raises the possibility that ERY-specific occupancy of GATA1 and TAL1 at these sites is KLF1-dependent.

### GATA1 and GATA2 regulate overlapping and divergent genetic programs in late megakaryopoiesis

Our data support the existence of a genome-wide GATA switch in MEG lineage primed genes. However, while GATA1 replaces GATA2 at an extensive set of bound CRMs, GATA2 continues to be expressed in late megakaryopoiesis (Supplemental Fig. 7B), which raises several questions: (1) Do GATA1 and GATA2 regulate common or distinct sets of target genes? (2) Does binding of these factors result in similar or different functional outcomes? and (3) What determines whether a GATA2-bound element in HSPCs will undergo a GATA switch in late megakaryopoiesis or remain occupied by GATA2?

**Figure 6.**
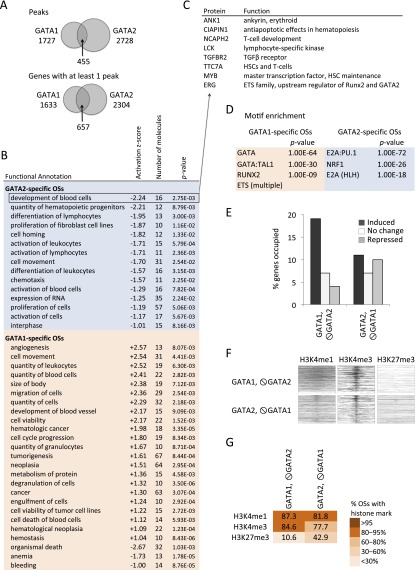
Distinct properties of GATA1 and GATA2 in late megakaryopoiesis. (*A*) Venn diagram showing the intersection of GATA1 and GATA2 occupancy peaks (Peaks) and bound genes (Genes) in MEG. (*B*) Ingenuity Pathway Analysis (IPA) showing predicted functions of genes associated with GATA1- or GATA2-specific chromatin OSs. A negative *Z*-score predicts mean repression of gene expression in the group, whereas a positive score predicts gene induction. Significantly enriched functional categories with ≥10 proteins and absolute *Z*-scores > 1, are shown. (*C*) Examples of GATA2-repressed genes in late megakaryopoiesis. (*D*) Motif enrichment in the 200-bp sequence centered on the transcription factor peaks at the GATA1- and GATA2-specific OSs, with those of the alternate GATA factor used reciprocally as background. (*E*) Fraction of up-regulated, unchanged, and down-regulated genes containing GATA1-selective and GATA2-selective OSs. (*F*) Heatmaps of H3K4me3, H3K4me1, and H3K27me3 histone modification marks centered around the transcription factor binding peaks at GATA1- and GATA2- specific OSs ordered from *top* to *bottom* by transcription factor peak significance. (*G*) Color-coded fractions of GATA1- and GATA2-specific transcription factor binding sites associated with H3K4me3, H3K4me1, and H3K27me3 histone methylation patterns in MEG.

We investigated GATA1 and GATA2 chromatin occupancy in primary cultured MEG, where both proteins are expressed simultaneously. We detected 2728 GATA2 binding sites mapping to 2304 genes across the MEG genome (Supplemental Fig. 3B). Only 455/2728 GATA2 OSs (< 17%) were shared by GATA1, suggesting unique binding preferences of these proteins for specific *cis* elements (Fig. 6A). Ingenuity Pathway Analysis (IPA) predicted enrichment of distinct biological functions for GATA1- versus GATA2-specific gene sets. In particular, the GATA2-occupied gene set was enriched for functions associated with maintenance of hematopoietic stem cells (HSCs) and development of alternate lineages; negative activation *Z*-scores predict that the genes representing these functional groups are repressed by GATA2 (Fig. 6B). For example, GATA2-mediated repression of *Myb* may contribute to the proliferation arrest that occurs in late megakaryopoiesis (Fig. 6C). In contrast, GATA1 was preferentially bound to activated genes that facilitate MEG/platelet function, such as those encoding platelet-derived growth factor, FLI1, and cell surface markers (Fig. 5; data not shown). Thus, in MEG, GATA1 and GATA2 regulate largely divergent genetic programs, in which GATA1 tends to activate MEG/platelet-specific genes while GATA2 tends to repress genes expressed by HSCs and alternate lineages. Furthermore, analysis of GATA1- and GATA2-specific OSs in MEG demonstrated different combinations of DNA motifs associated with the two factors (Fig. 6D). Thus, whether a GATA2-bound CRM in HSPCs will undergo a GATA switch during MEG differentiation or remain GATA2-bound likely depends on cobinding of additional proteins such as TCF3, NRF1, or ETS factors.

Roughly equal proportions of genes bound specifically by GATA2 during MEG development were activated, repressed, and unchanged compared to their expression in the HSPC population (Fig. 6E). Accordingly, GATA2-specific MEG OSs demonstrated a fourfold higher enrichment for the repressive H3K27me3 histone methylation mark compared with the GATA1-specific OSs, which occurred preferentially within up-regulated genes (Fig. 6E–G; Supplemental Figs. 14, 15). This might be related to the presence of ETS elements specifically adjacent to GATA1 binding sites (Fig. 6D), which is associated with gene activation. Overall, our data indicate that compared to GATA1, GATA2 plays a more pronounced role as a transcriptional repressor, regulating distinct sets of genes during MEG differentiation.

## Discussion

**Figure 7.**
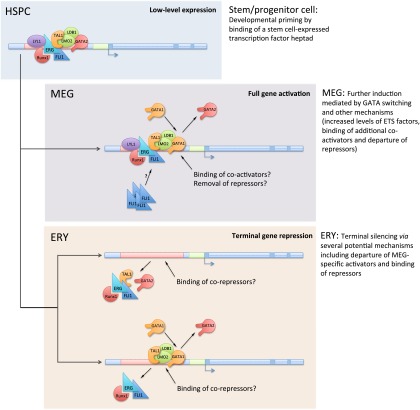
A model for developmental regulation of gene expression during erythro-megakaryopoiesis. A stem/progenitor cell-expressed transcription factor “heptad” (GATA2, LYL1, TAL1, ERG, FLI1, RUNX1, and LMO2) binds and transcriptionally primes MEG-specific genes in hematopoietic stem/progenitor cells (HSPCs) (*top*). Events that occur upon subsequent differentiation of these cells into MEG and ERY are indicated in the *middle* and *bottom* panels. Further transcriptional activation of the primed genes in MEG is mediated by GATA switching (replacement of GATA2 by GATA1), ETS factors, and other mechanisms. In most cases, terminal silencing of primed MEG-specific genes in ERY depends on the departure of GATA proteins and MEG-specific transcriptional activators, such as ETS factors, and possible binding of ERY-specific transcriptional repressors. In some instances, terminal repression of MEG-specific genes in ERY appears to be associated with GATA switching and likely relies on recruitment of ERY-specific transcriptional repressors. In addition to the heptad of factors identified in HPC-7 cells (Wilson et al. 2010), the lineage priming “heptad” also likely includes the protein LDB1, which mediates interactions among several of the proteins.

We used global transcriptome profiling and ChIP-seq to define and compare DNA occupancy by hematopoietic transcription factors, histone modifications, and mRNA expression during erythro-megakaryocytic differentiation. Integrating our findings with prior studies establishes a more coherent, genome-wide model for MEG and ERY-MEG lineage priming in HSPCs (Wilson et al. 2010). According to this model, a HSPC-expressed transcription factor heptad (GATA2, LYL1, TAL1, ERG, FLI1, RUNX1, and LMO2) binds and transcriptionally primes an extensive set of MEG-specific (as well as some common ERY-MEG) genes during early hematopoiesis (Fig. 7). The principal components of this transcription factor complex, ETS and GATA factors along with TAL1, remain bound to their CRMs while the gene undergoes further transcriptional activation following lineage commitment. Importantly, a GATA switch occurs at most of these loci, suggesting that this mechanism regulates in part the transition from low-level expression in HSPCs to full induction after lineage commitment, particularly in MEG. In agreement, GATA switch sites are preferentially associated with genes undergoing transcriptional activation (Fig. 2E). Many of the genes primed in HSPCs and induced in MEG via this mechanism are silenced in ERY and are enriched for ETS factor binding motifs. Although it is unclear whether additional repressors are recruited to silence the MEG-primed genes in ERY, it is likely that the reduction of ETS factors following ERY commitment and maturation contributes to the departure of GATA and TAL1 at most MEG-specific loci (Figs. 5A, 7). Our data therefore confirm that ETS factors play a critical role in MEG lineage specification and divergence from ERY and indicate a new role for ETS factors in MEG-specific lineage priming in HSPCs. In addition, our study provides insights into the mechanisms of GATA1 and TAL1 lineage binding selectivity. A more complex network of potential GATA1- and TAL1-associated factors than previously appreciated emerges from our discriminative motif enrichment approach and our kmer-SVM analysis.

Up to 40% of MEG GATA1 OSs are bound by GATA2 in HSPCs. This resembles the proportion of GATA1 OSs that were shown recently to undergo a GATA1 switch in the erythro-megakaryocytic cell line G1ME (Dore et al. 2012), although the absolute number of MEG GATA1 OSs detected in the current study was several fold reduced. This may be due to technical differences between the two studies, including sensitivities of the antibodies used or peak calling algorithms. Alternatively, the true number of MEG GATA switch sites may be overestimated in the G1ME system, where GATA1 expression is driven by a retrovirus and not endogenous regulatory elements. Regardless, both studies confirm the existence of a genome-wide GATA factor switch in MEG development. Notably, although the GATA switch sites appear to participate equally in gene repression and activation in G1ME cells (Dore et al. 2012), we find that most GATA switch sites detected in primary cultured MEG are associated with developmental gene induction and are enriched for TAL1 binding. Consistent with a primary role in gene activation, GATA switch sites are enriched for H3K4me1 and H3K4me3 and depleted for H3K27me3 histone methylation marks. Thus, GATA switching should be considered in the context of genome-wide hematopoietic lineage priming by GATA2-containing complexes and represents a mechanism of terminal lineage-specific activation of developmentally primed genes. This mechanism may also occur in other GATA-1-regulated hematopoietic lineages, such as mast cells, dendritic cells, and eosinophils.

Our data demonstrate that GATA1-TAL1 association distinguishes GATA1-mediated gene activation in MEG, similar to what is reported in ERY (Cheng et al. 2009; Tripic et al. 2009; Yu et al. 2009). Co-occupancy by GATA1 and FLI1 is also strongly associated with gene induction in MEG, thus confirming a previously proposed model on a genome-wide scale (Wang et al. 2002). Notably, other ETS factors, including ETS1 and GABPA, have also been shown to associate with GATA1 in megakaryopoiesis (Pang et al. 2006; Dore et al. 2012). Additional genome-wide studies would expand our understanding of their developmental roles and functional divergence.

Persistence of GATA2 in mature MEG contrasts with the loss of GATA2 in ERY (Bresnick et al. 2010). Remarkably, GATA1 and GATA2 regulate largely distinct gene sets in MEG, with strikingly different functional roles and *cis*-element binding motifs. Thus, although GATA1 and GATA2 can partially compensate for one another in gene targeting experiments (Blobel et al. 1995; Tsai et al. 1998; Fujiwara et al. 2004; Ferreira et al. 2007), functional divergence between these two related transcription factors is apparent in MEG development. Motif analysis predicts that cobinding of NRF and TCF3 contribute to the GATA2 binding specificity and may prevent GATA switching at GATA2-specific MEG CRMs. In contrast, ETS binding motifs at GATA CRMs are associated with GATA switching, although a full set of GATA1 versus GATA2 binding requirements at various stages of MEG development remains to be elucidated. Compared to GATA1, GATA2 appears to catalyze preferentially a more extensive program of gene repression associated with H3K27me3 methylation near bound sites. Overall, our findings suggest a distinct role for GATA2 in the repression of HSPC- and alternate lineage-expressed genes in MEG, whereas GATA1 appears to regulate lineage-specific gene activation.

Our experimental approach included large-scale, genome-wide surveillance of developmental gene regulation during erythro-megakaryopoiesis. However, there are some intrinsic limitations imposed by various technical issues. First, it was necessary to generate MEGs from cultured primary fetal liver progenitors in order to obtain sufficient quantities for ChIP-seq to detect chromatin-bound transcription factors. Although cultured MEGs may differ somewhat from primary MEGs, they are likely more representative than immortalized cell lines. Second, fetal and adult hematopoiesis exhibit small but biologically important differences, posing potential limitations for extrapolating our results obtained from fetal-derived cells to adult tissues (Sola-Visner 2012; Xu et al. 2012). Third, the scarcity of endogenous HSPCs and the inability to expand them ex vivo necessitated the use of ChIP-seq data generated in a surrogate cell line (HPC-7). Finally, our bioinformatics studies utilized the common simplifying assumption that TF-bound CRMs regulate the gene that they are closest to. Although it is well appreciated that some enhancers act at very long distances, even “skipping” the nearest gene (Li et al. 2012; Sanyal et al. 2013; Smallwood and Ren 2013), high-throughput techniques to accurately link such distant CRMs to their targets are only now being developed. It is also clear that much regulatory information lies within or close to genes. Genes responsive to hormones or to GATA1 tend to have factor-bound DNA segments nearby (Cheng et al. 2009; Reddy et al. 2009). Furthermore, most expression quantitative trait loci are near to the genes that they regulate (Gilad et al. 2008). In a separate study that includes the data sets described here to analyze TAL1-regulated gene expression in hematopoiesis, we obtained similar results by assigning CRM targets as the most proximal gene and by using an alternative approach that defined “enhancer-promoter units (EPUs)” to predict CRM target genes independent of gene proximity (Shen et al. 2012; Wu et al. 2014). More accurate methods to pair CRMs with their target genes will further refine the functional correlations identified in this study, but will not likely change the main conclusions.

Despite these unavoidable limitations, we reveal a global transcriptional mechanism of hematopoietic lineage priming extending from HSPC to the MEG lineage, reflecting divergent, lineage-specific functions for the common transcription factors GATA1, GATA2, TAL1, and FLI1 (see also Wu et al. 2014). We also extend the concept of genome-wide MEG GATA switching, with unique features compared to ERY GATA switching. Our data indicate that early hematopoietic progenitors may be biased toward megakaryopoiesis by preferential priming of MEG lineage-specific genes. Indeed, a stable population of “platelet-biased” stem cells residing at the apex of the HSC developmental hierarchy was recently identified (Sanjuan-Pla et al. 2013). Our data support and extend these findings by indicating a potential transcriptional mechanism for such bias. In contrast, erythropoiesis appears to involve a more fundamental rewiring of MEG-primed stem cell circuits, likely by ERY-specific transcription factors. For example, aberrant expression of MEG genes occurs in KLF1-null erythroblasts, suggesting that KLF1 inhibits the MEG-specific developmental program during erythropoiesis (Bouilloux et al. 2008; Siatecka et al. 2010; Tallack and Perkins 2010).

Recent efforts have produced genome-wide transcription factor occupancy maps for a variety of organisms and tissues (The ENCODE Project Consortium 2012; The Mouse ENCODE Consortium et al. 2012; The Mouse ENCODE Consortium et al. 2014). Decoding this extensive set of information to better understand tissue development and function represents a significant challenge. The current study enhances our understanding of blood development by functional analysis of the Mouse ENCODE Consortium genome-wide transcription factor occupancy data in a specific developmental context, namely the divergence of ERY and MEG lineages from a common progenitor. As expected, the benefit of the data generated within this consortium was enhanced by generating additional data, such as the transcriptome microarrays, and incorporating data from other studies, such as those on HPC-7 cells (Wilson et al. 2010). An integrative analysis of data from all these sources was critical to reach our conclusions. More generally, our data illustrate new mechanisms by which largely overlapping sets of transcription factors orchestrate the development of two hematopoietic lineages via divergent combinatorial interactions acting at distinct *cis*-elements. Similar concepts likely apply to the transcriptional control of cellular differentiation in other tissues.

## Methods

### Purification of mouse fetal hematopoietic cells

Mouse fetal livers (E14.5) were used as the source of primary cells (Fig. 1; Supplemental Fig. 1), as described by Zhang et al. 2003. For purification of HSPC, fetal liver cell suspension was stained with biotinylated antibodies against lineage antigens (CD5, CD11b, CD19, CD45R, Ly-6G/C, Ter119, CD71). Lineage-positive cells were removed using a magnetic bead cell separation protocol and discarded. The lineage-negative cells were stained with an APC-conjugated anti-Sca1 antibody followed by purification of Sca1+ cells by FACS sorting. Expression levels of three endothelial cell markers, *Chd5* (CD144), *Thbd* (CD141), and *Plvap* (MECA32), were below the 10th percentile of expressed genes (7.93%, 8.77%, and 6.33%, respectively), indicating no significant contamination of the purified HSPC population by endothelial cells. Primary erythroblasts (ERY) were obtained by immunostaining of fetal liver cells with PE-conjugated antibodies against Ter119 followed by magnetic bead purification. Primary fetal liver megakaryocytes were obtained by expansion and differentiation of fetal liver hematopoietic progenitor cells. KIT-positive progenitors were isolated directly from E14.5 mouse fetal livers by immunomagnetic selection and expanded in a medium with SCF and TPO for 7 d followed by terminal megakaryocyte differentiation for 5 d with TPO only. Differentiated megakaryocytes were further enriched to >97% purity by CD41 immunomagnetic bead selection. All cell populations were purified to at least 94% purity.

### Transcriptome analysis

mRNA was extracted using standard affinity purification techniques and analyzed on GeneChip Mouse Gene 1.0 ST Arrays with four biological replicates for each cell type. We used the HSPC transcriptome as a reference point to define the developmental changes in gene expression during monolineage differentiation (Supplemental Methods). For MEG versus HSPC and ERY versus HSPC comparisons, genes that significantly (FDR < 5%) changed more than twofold relative to the expression level in HSPC were considered to be developmentally up- or down-regulated. Genes whose expression was altered insignificantly (nominal *P* > 0.05) or less than 1.2-fold were considered to be unchanged (Fig. 1E). Genes that changed between 1.2 and twofold were considered indeterminable and not examined further in this study. As an independent assessment of cell purity, we confirmed differential expression of selected lineage-enriched genes by TaqMan RT-PCR (Supplemental Fig. 7B).

### Chromatin immunoprecipitation and massively parallel DNA sequencing

ChIP-seq was performed with the use of standardized ENCODE procedures, as previously described (Supplemental Methods; Cheng et al. 2009; Landt et al. 2012). We used the approach of irreproducible discovery rate at a threshold of 0.02 to determine the number *n* of reproducible peaks in the replicate data sets (Li et al. 2011; Landt et al. 2012). Peaks were called on the combined reads from all replicates and the top *n* peaks were taken as the set of high confidence peaks. This is a very conservative method for thresholding, and we also generated a larger set of quality peaks, called the reduced stringency peaks, by applying a threshold based on the *P*-value of the least significant peak in the top 90% of reproducible peaks (the threshold *P*-values ranged from 10^−80^ to 10^−160^; a detailed description is presented in Supplemental Methods). The high stringency set of peaks was used for all analyses, and for cases in which the smaller number of peaks could affect the interpretation, the analysis was repeated for the larger set of reduced stringency peaks. A detailed description of experimental protocols and statistical analysis of the data is presented in Supplemental Methods.

## Data access

Mapped sequencing reads and cDNA microarray data have been submitted to the NCBI Gene Expression Omnibus (GEO; http://www.ncbi.nlm.nih.gov/geo) under accession number GSE49664. Reads, peak calls, and signal tracks are also available from our customized genome browser (http://main.genome-browser.bx.psu.edu/), the UCSC Genome Browser (http://genome.ucsc.edu/), and the Mouse ENCODE Consortium website (http://www.mouseencode.org/publications/mcp08/).

## Supplementary Material

Supplemental Material
