# Supplementary material for: Divergent functions of hematopoietic transcription factors in lineage priming and differentiation during erythro-megakaryopoiesis
Source: Genome Res. 2014 Dec;24(12):1932–44. doi: 10.1101/gr.164178.113 (PMC4248311; doi:10.1101/gr.164178.113)
Supplement: Supplemental Material [file supp_24_12_1932__index.html]

Divergent functions of hematopoietic transcription factors in lineage priming and differentiation during erythro-megakaryopoiesis — Divergent functions of hematopoietic transcription factors in lineage priming and differentiation during erythro-megakaryopoiesis — Supplemental Material 

# Divergent functions of hematopoietic transcription factors in lineage priming and differentiation during erythro-megakaryopoiesis

## Supplemental Material

**Files in this Data Supplement:**

- Supplemental Figures.pdf
- Supplemental Methods.pdf
- Supplemental Tables.pdf
