# Supplementary material for: Divergent functions of hematopoietic transcription factors in lineage priming and differentiation during erythro-megakaryopoiesis
Source: Genome Res. 2014 Dec;24(12):1932–44. doi: 10.1101/gr.164178.113 (PMC4248311; doi:10.1101/gr.164178.113)
Supplement: Supplemental Material [file supp_gr.164178.113_Supplemental_Figures.pdf]

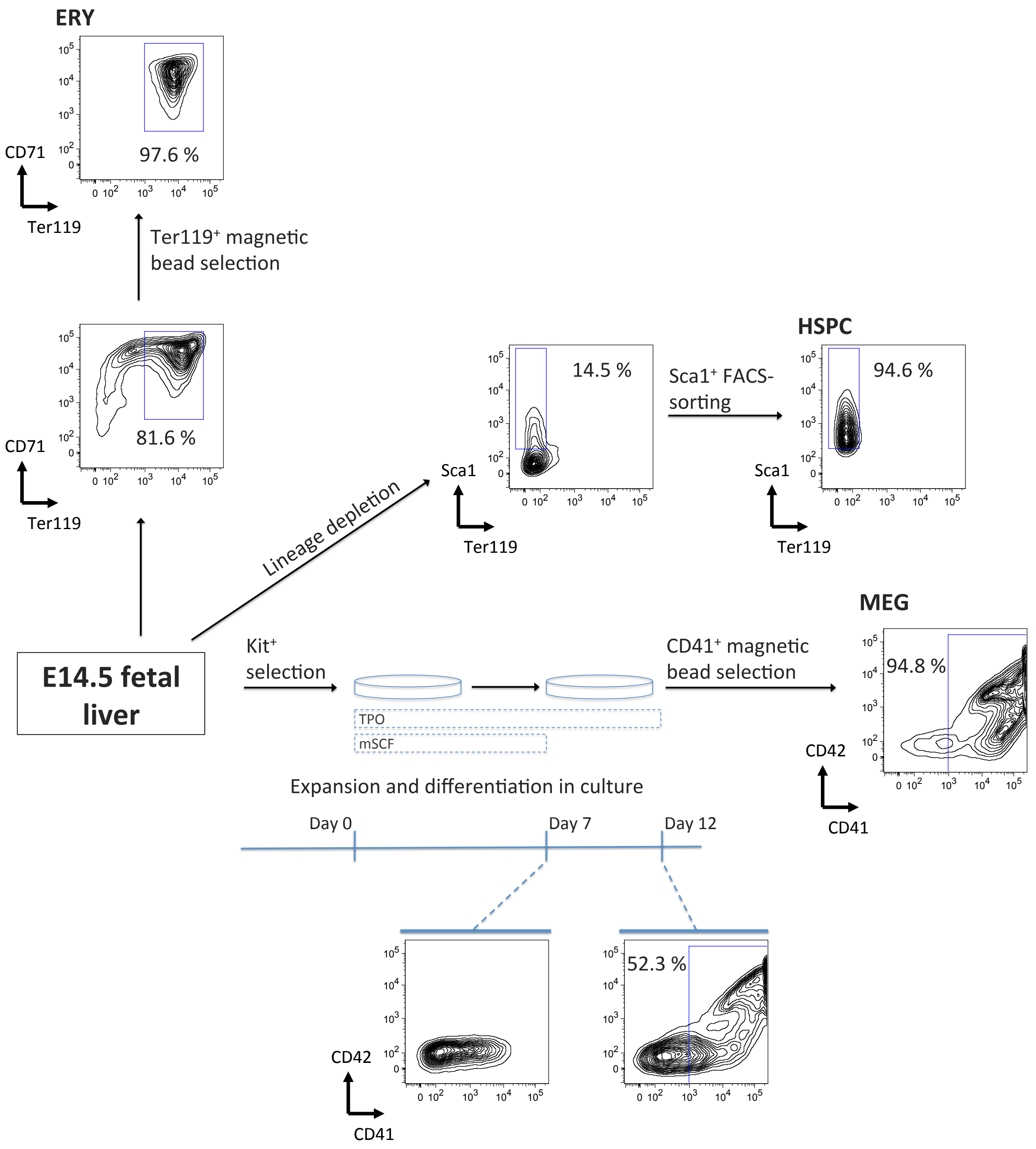

Supplemental Figure 1. Methods for purifying erythroblasts (ERY), megakaryocytes (MEG) and hematopoietic stem/progenitor cells (HSPCs). Flow cytometry density plots of intermediate and final cell populations are shown, stained with anti-cell surface marker antibodies as indicated.

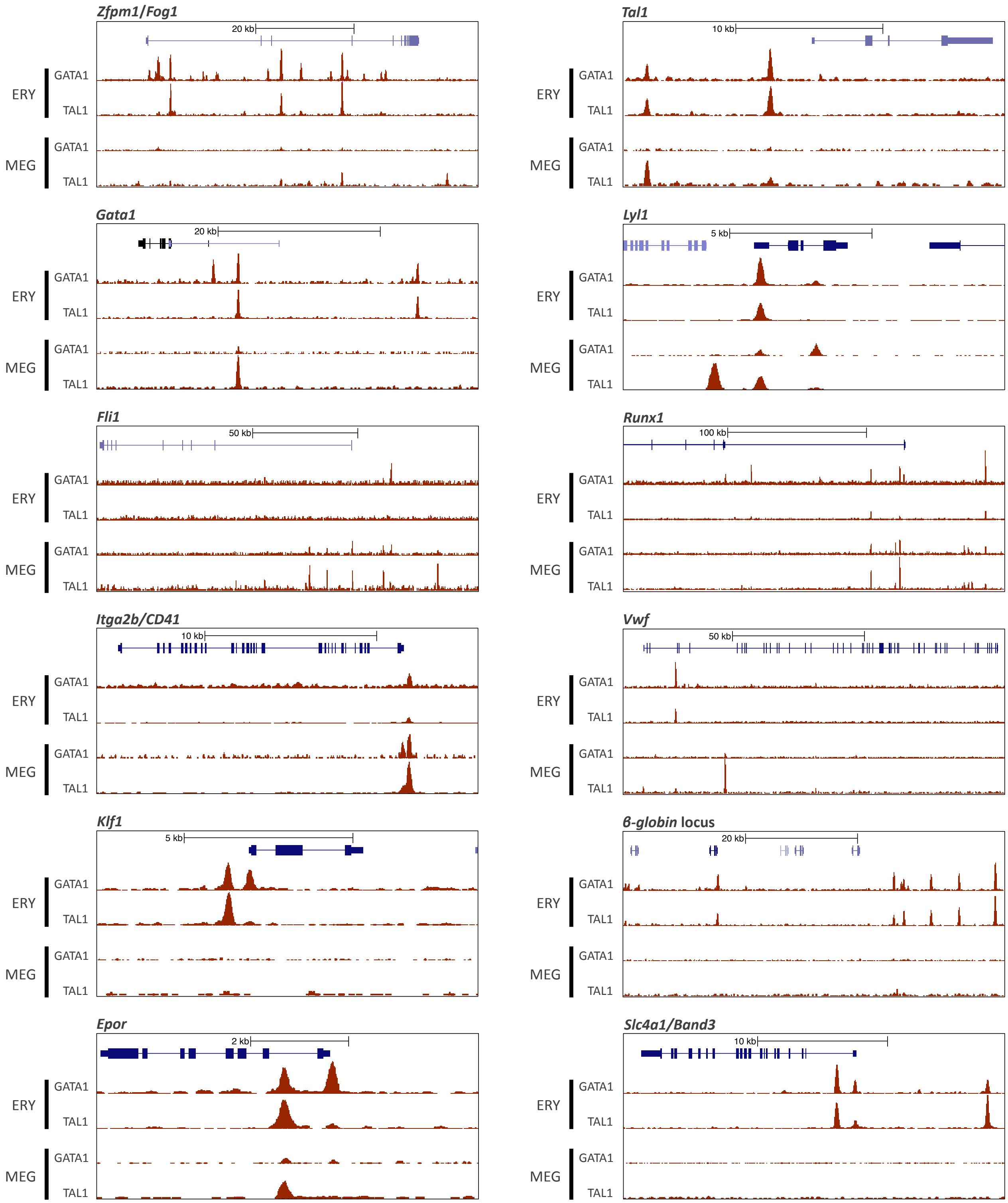

Supplemental Figure 2. Examples of genes with lineage-specific patterns of chromatin occupancy. Occupancy of GATA1 and TAL1 in ERY and MEG, as determined by ChIP-seq, are shown below a schematic of each gene.

A

		ERY		MEG			
		GATA1	TAL1	GATA1	GATA2	TAL1	FLI1
ERY	GATA1	-	2711	433	-	-	-
	TAL1	2711	-	-	-	242	-
MEG	GATA1	433	-	-	455	757	316
	GATA2	-	-	455	-	342	197
	TAL1	-	242	757	342	-	375
	FLI1	-	-	316	197	375	-

B

		Total # of peaks	% peaks in genes	Genes with at least 1 peak	Expressed genes with at least 1 peak
ERY	GATA1	5767	77%	5116	2411
	TAL1	3086	75%	2848	1352
MEG	GATA1	1727	66%	1633	765
	GATA2	2728	63%	2304	863
	TAL1	3505	62%	2840	1149
	FLI1	2001	84%	3013	1542

Supplemental Figure 3. Characterization of genome-wide transcription factor occupancy. *A*, Intersection of transcription factor binding peaks within and between lineages. *B*, Numbers and distribution of transcription factor binding peaks across the genome.

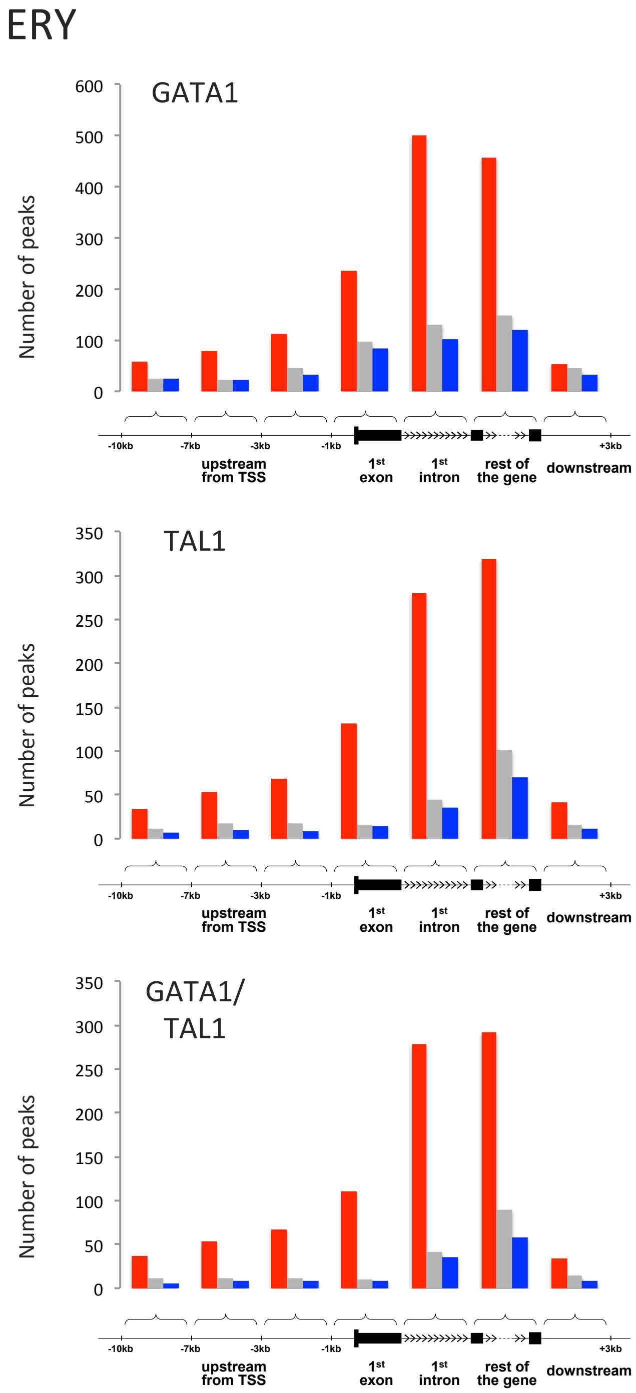

Supplemental Figure 4. Distribution of transcription factor OSs across gene structure and its correlation with the developmental gene expression response in ERY. Distribution of GATA1 and TAL1 OSs across the gene structure, shown as the absolute number of peaks called in a given gene segment, in up-regulated (red), down-regulated (blue) and non-responsive (grey) gene categories. Exons are indicated by black rectangles. Introns are represented by thin lines with arrows marking the direction of transcription.

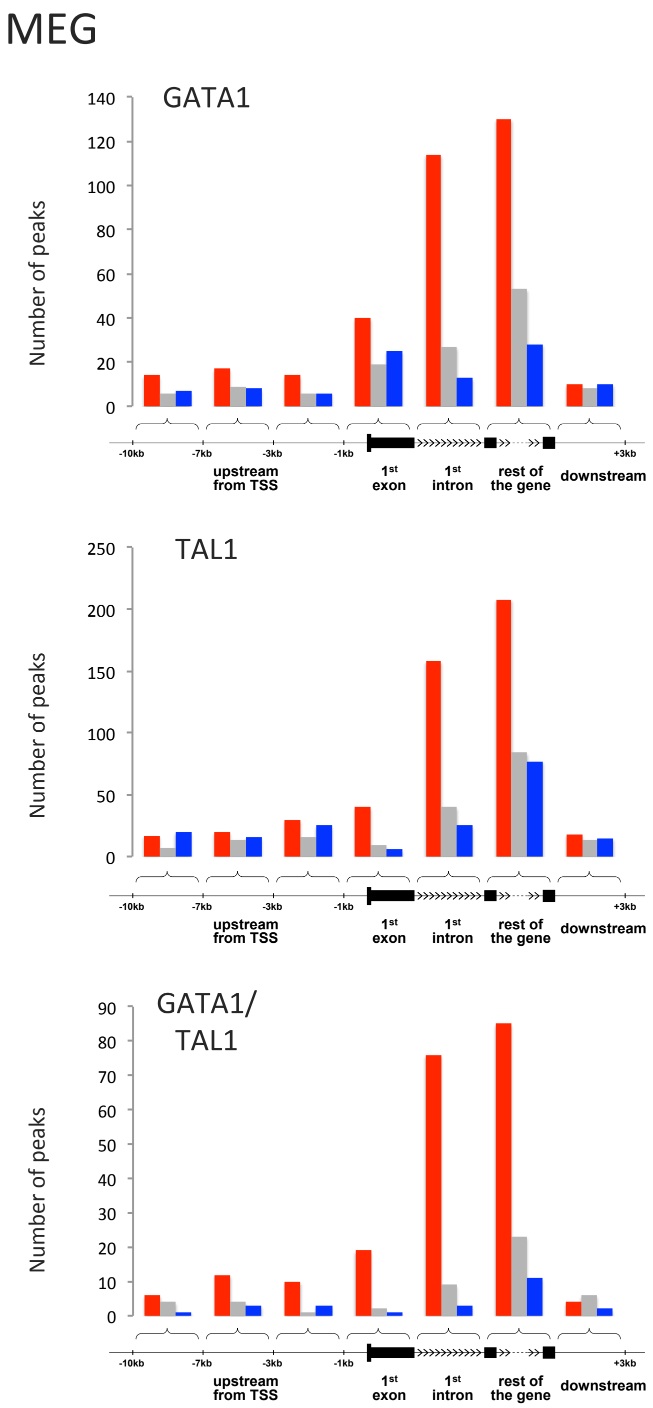

Supplemental Figure 5. Distribution of transcription factor OSs across gene structure and its correlation with the developmental gene expression response in MEG. Distribution of GATA1 and TAL1 occupancy across the gene structure, shown as the absolute number of peaks called in a given gene segment, in up-regulated (red), down-regulated (blue) and non-responsive (grey) gene categories. Introns are represented by thin lines with arrows marking the direction of transcription.

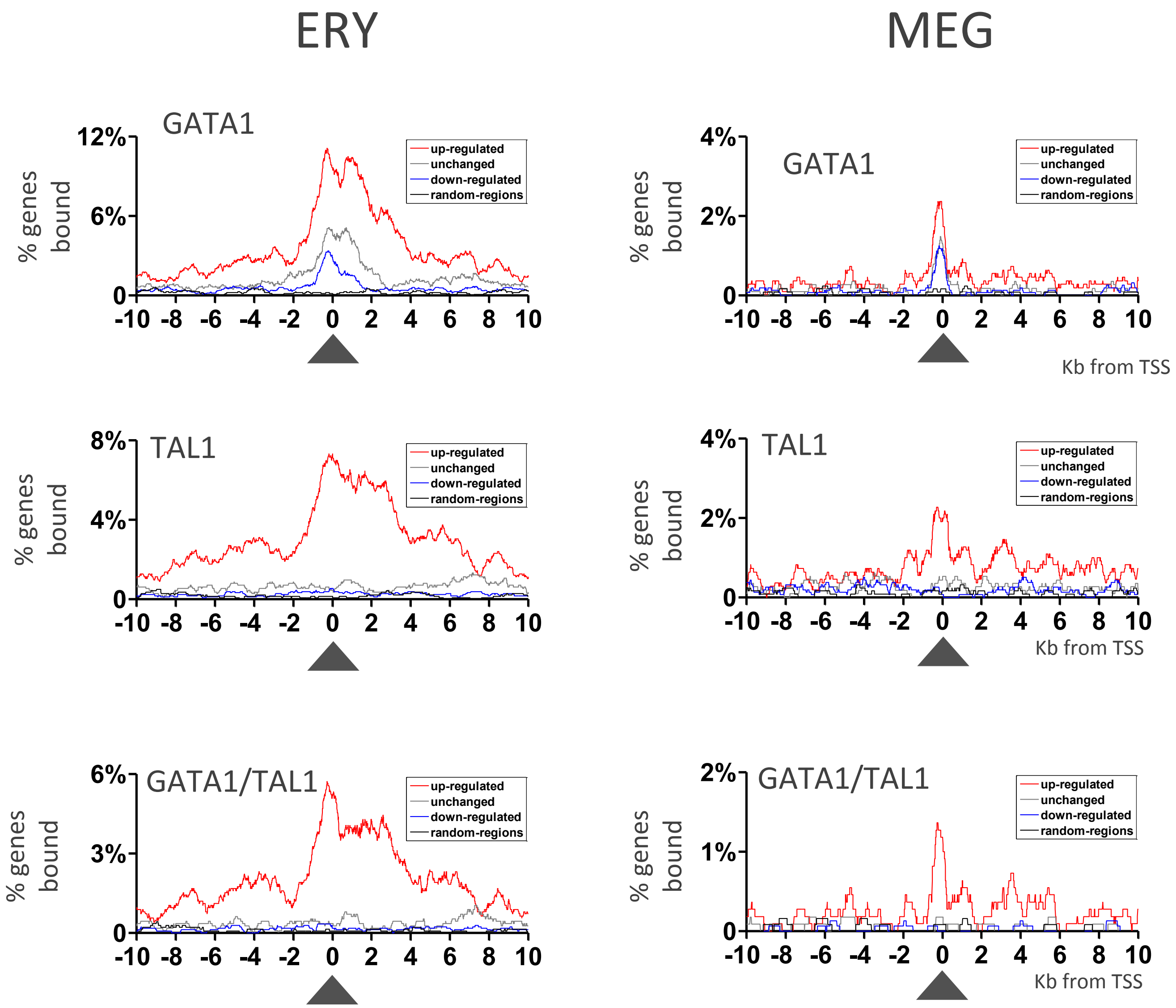

Supplemental Figure 6. Distribution of GATA1 and TAL1 OSs relative to TSS (indicated by black arrows) in up-regulated (red), down-regulated (blue) and non-responsive (grey) genes. Horizontal axis, Kb upstream or downstream from TSS (arrow). Vertical axis, % genes in a given response category with at least one transcription factor binding peak.

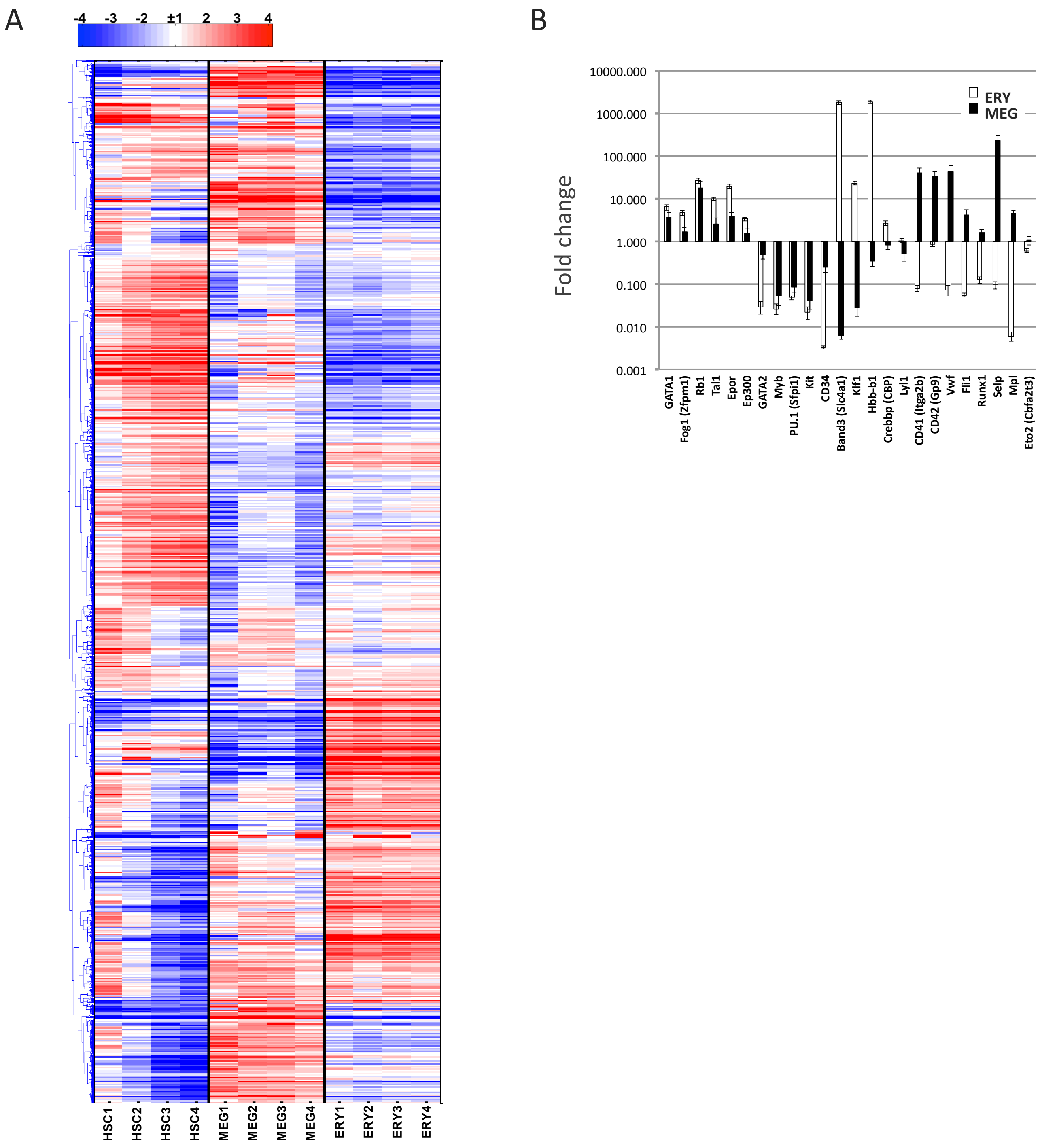

Supplemental Figure 7. Messenger RNA expression during erythro-megakaryopoiesis. *A*, Global hierarchical clustering of the 7513 expressed genes in four biological replicates based on Spearman correlation distance. *B*, Developmental changes in mRNA expression in ERY and MEG relative to HSPCs defined by TaqMan PCR for selected genes.

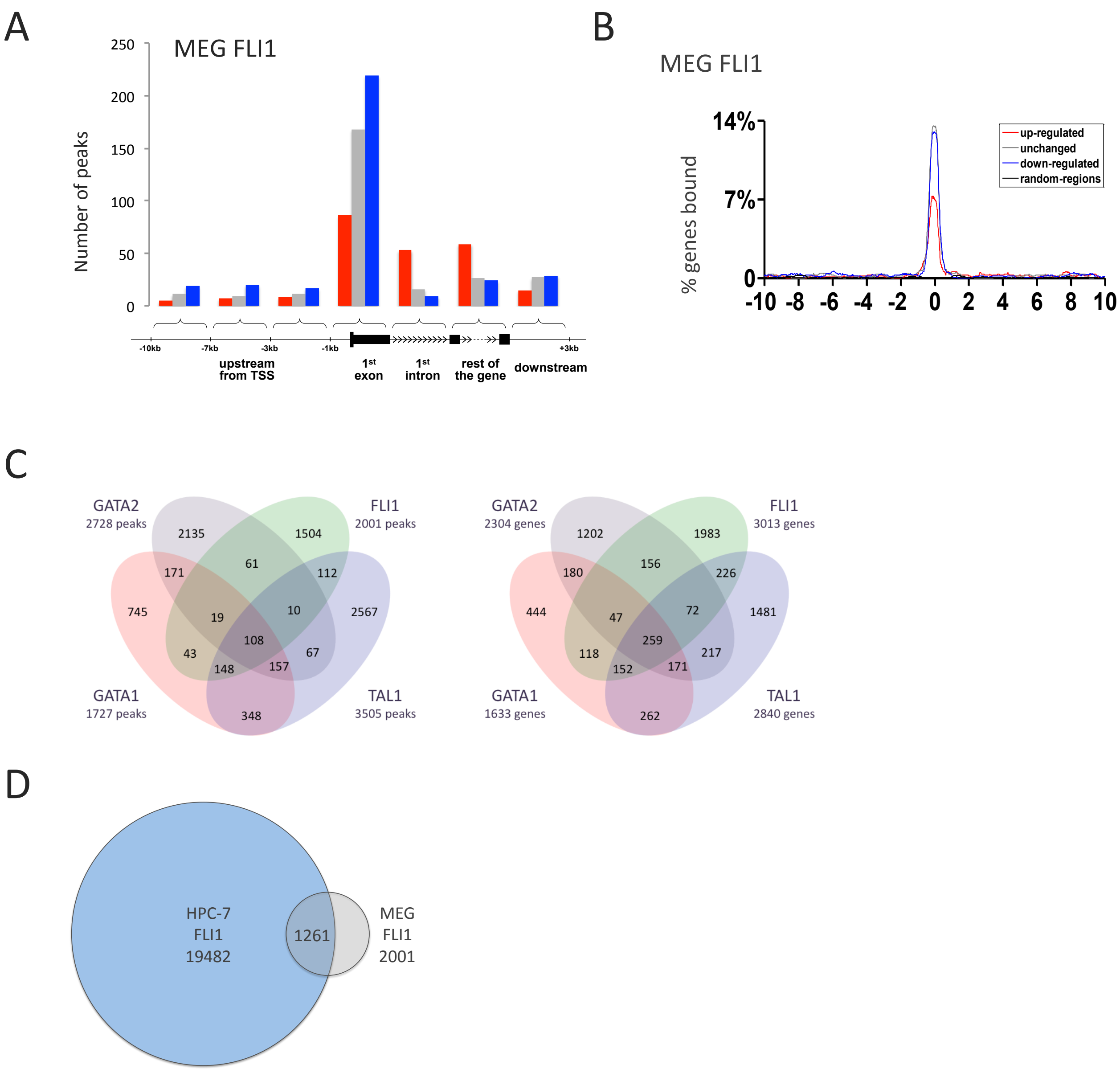

Supplemental Figure 8. FLI1 occupancy in MEG. *A*, Distribution of FLI1 OSs across the gene structure, shown as the absolute number of peaks called in a given gene segment, in up-regulated (red), down-regulated (blue) and non-responsive (grey) gene categories. Introns are represented by thin lines with arrows marking the direction of transcription. *B*, Distribution of FLI1 OSs relative to TSS (indicated by black arrows) in up-regulated, down-regulated and non-responsive gene categories. *Horizontal axis*, Kb from upstream or downstream from TSS (arrow). *Vertical axis*, % genes in a given response category with at least one transcription factor binding peak. *C*, A comprehensive intersection of GATA1, GATA2, TAL1 and FLI1 occupancy in MEG, for specific occupancy peaks (left) and genes (right). *D*, Intersection of FLI1 binding peaks in MEG *versus* HPC-7 cells (Wilson et al., 2010).

% MEG/ERY transcription factor peaks overlapping with HPC-7 peaks

HPC7	MEG	ERY	N HPC7	N MEG/ERY	N overlapping	% overlap
ERG		GATA1/TAL1	35120	2711	**661**	24.4%
ERG		GATA1	35120	5767	**1596**	27.7%
ERG		TAL1	35120	3086	**812**	26.3%
ERG	GATA1/TAL1		35120	757	**559**	73.8%
ERG	GATA1		35120	1727	**954**	55.2%
ERG	TAL1		35120	3505	**1214**	34.6%
ERG	FLI1		35120	2001	**1776**	88.8%
ERG	GATA2		35120	2728	**820**	30.1%
Fli1		GATA1/TAL1	19482	2711	**445**	16.4%
Fli1		GATA1	19482	5767	**863**	15.0%
Fli1		TAL1	19482	3086	**520**	16.9%
Fli1	GATA1/TAL1		19482	757	**565**	74.6%
Fli1	GATA1		19482	1727	**888**	51.4%
Fli1	TAL1		19482	3505	**1122**	32.0%
Fli1	FLI1		19482	2001	**1283**	64.1%
Fli1	GATA2		19482	2728	**760**	27.9%
Gata2		GATA1/TAL1	9120	2711	**450**	16.6%
Gata2		GATA1	9120	5767	**895**	15.5%
Gata2		TAL1	9120	3086	**493**	16.0%
Gata2	GATA1/TAL1		9120	757	**420**	55.5%
Gata2	GATA1		9120	1727	**684**	39.6%
Gata2	TAL1		9120	3505	**669**	19.1%
Gata2	FLI1		9120	2001	**273**	13.6%
Gata2	GATA2		9120	2728	**732**	26.8%
Lmo2		GATA1/TAL1	9518	2711	**706**	26.0%
Lmo2		GATA1	9518	5767	**899**	15.6%
Lmo2		TAL1	9518	3086	**781**	25.3%
Lmo2	GATA1/TAL1		9518	757	**583**	77.0%
Lmo2	GATA1		9518	1727	**805**	46.6%
Lmo2	TAL1		9518	3505	**1144**	32.6%
Lmo2	FLI1		9518	2001	**432**	21.6%
Lmo2	GATA2		9518	2728	**685**	25.1%
Lyl1		GATA1/TAL1	4326	2711	**312**	11.5%
Lyl1		GATA1	4326	5767	**395**	6.8%
Lyl1		TAL1	4326	3086	**339**	11.0%
Lyl1	GATA1/TAL1		4326	757	**383**	50.6%
Lyl1	GATA1		4326	1727	**487**	28.2%
Lyl1	TAL1		4326	3505	**663**	18.9%
Lyl1	FLI1		4326	2001	**254**	12.7%
Lyl1	GATA2		4326	2728	**456**	16.7%
PU1		GATA1/TAL1	21664	2711	**226**	8.3%
PU1		GATA1	21664	5767	**673**	11.7%
PU1		TAL1	21664	3086	**297**	9.6%
PU1	GATA1/TAL1		21664	757	**185**	24.4%
PU1	GATA1		21664	1727	**423**	24.5%
PU1	TAL1		21664	3505	**438**	12.5%
PU1	FLI1		21664	2001	**1080**	54.0%
PU1	GATA2		21664	2728	**629**	23.1%
Runx1		GATA1/TAL1	5202	2711	**344**	12.7%
Runx1		GATA1	5202	5767	**473**	8.2%
Runx1		TAL1	5202	3086	**384**	12.4%
Runx1	GATA1/TAL1		5202	757	**414**	54.7%
Runx1	GATA1		5202	1727	**560**	32.4%
Runx1	TAL1		5202	3505	**740**	21.1%
Runx1	FLI1		5202	2001	**402**	20.1%
Runx1	GATA2		5202	2728	**462**	16.9%
SCL/TAL1		GATA1/TAL1	7083	2711	**529**	19.5%
SCL/TAL1		GATA1	7083	5767	**818**	14.2%
SCL/TAL1		TAL1	7083	3086	**590**	19.1%
SCL/TAL1	GATA1/TAL1		7083	757	**499**	65.9%
SCL/TAL1	GATA1		7083	1727	**657**	38.0%
SCL/TAL1	TAL1		7083	3505	**976**	27.8%
SCL/TAL1	FLI1		7083	2001	**773**	38.6%
SCL/TAL1	GATA2		7083	2728	**492**	18.0%

Supplemental Figure 9. Intersection of HPC-7 transcription factor OSs with MEG and ERY. Absolute numbers of overlapping peaks ("*N overlapping*"), as well as fraction of MEG/ERY peaks ("*% overlap*") that overlap with HPC-7 peaks, are shown. Fractions of overlapping peaks are color-coded according to percent overlap (darker shading corresponds to greater overlap). For example, as seen in row 5, 73.8 % of MEG GATA1/TAL1 co-occupant OSs overlap with (i.e. occupy the same enhancers) as ERG in HPC-7 cells.

A

	MEG vs HSPC	ERY vs HSPC	# of genes	self-renewal of stem cells	ES cell death	myeloproliferative disorder	lymphocyte morphology and differentiation	leukocyte function	quantity of leukocytes	quantity of blood/bone marrow cells	quantity of RBCs/anemia	enucleation of RBC	porphyrin metabolism	catabolism of hydrogen peroxide	hemostasis	platelet activation/aggregation	angiogenesis	lipid transport	conformational modification of DNA	exit from M phase	apoptosis of blood cells	expression of mRNA	translation of mRNA	processing of rRNA	protein metabolism	apoptosis	protein catabolism	carbohydrate synthesis/metabolism	Top enriched motifs in promoter areas	Enriched upstream regulators
1	U	U	380		■					■	■	■														■	■	■	KLF4, AP1, NF-E2, GATA:SCL	NRF2, FOS, HIPK, GATA
2	D	D	898	■		■	■	■																■					c-Myc, ETS	IL4, PTPN11, EIF4E
3	U	D	134						■	■					■	■													ETS, PRDM9, GATA:SCL	IL4, KLF1, PLN, ZDHHC2
4	D	U	93								■		■		■			■											HNF1, FOXA1/2, MyoD	PPARA, HNF1A/B, FOXA2
5	U	N	171													■	■											■	GRE, FOXP1, HOXB4	IL5, ESRRA, SerpinD, ETS1
6	D	N	121																■	■									E2F, NFY, YY1, FOXA1, ETS	E2F1, E2F4, EP400, RB1
7	N	U	181											■															Sp1, NRF1	miR-483, BNIP3L, EP400
8	N	D	173				■	■	■												■								ETS, ESRRB, STAT6	HLX, ESRRA, AZGP1, LYN
9	N	N	280																			■	■		■				ETS, NRF1	NRF2, ATF1, HSF2, FLI1

(Columns "self-renewal of stem cells" through "carbohydrate synthesis/metabolism" are grouped under the heading "Function enrichment".)

B

		Motif	p-value
**Concordant (Group 1, UU)**			
	GATA1 OSs	n-MYC (HLH)	1.00E-04
		FOXA2	1.00E-04
		c-MYC (HLH)	1.00E-03
	GATA1 OSs, ⃠FLI1	MAFK	1.00E-05
		n-MYC (HLH)	1.00E-04
		c-MYC (HLH)	1.00E-03
		FOXA2	1.00E-03
**Discordant (Group 3, UD)**			
	GATA1 OSs	ERG (ETS)	1.00E-10
		ELK1 (ETS)	1.00E-09
		ETV1 (ETS)	1.00E-06
		ETS1 (ETS)	1.00E-05
		FLI1 (ETS)	1.00E-05
		PRDM1	1.00E-04
	GATA1 OSs, ⃠FLI1	ERG (ETS)	1.00E-08
		MyoG (HLH)	1.00E-05
		ELK1 (ETS)	1.00E-04
		ETV1 (ETS)	1.00E-04
		TCF12 (HLH)	1.00E-04
		ETS1 (ETS)	1.00E-04

Supplemental Figure 10. Analysis of genes according to their developmental expression patterns. *A*, Ingenuity Pathway Analysis (IPA) showing predicted functions for genes within each cluster. Also shown are predictions for enriched transcription factor binding motifs (against random background) within "promoter areas", defined as -2000 to +500 bp around the TSS, and upstream regulators. Clustering of genes is based on the combined directionality of developmental expression changes in MEG *versus* ERY with HSPCs as a reference starting point. "*U*", up-regulated. "*D*", down-regulated. "*N*", no change. See also Fig. 3 in the main text. *B*, Motifs enriched in the 200-bp sequence centered on the transcription factor peaks at the GATA1 OSs in gene cluster 1 (up-regulated in MEG and ERY) versus cluster 3 (up-regulated in MEG, down-regulated in ERY) with GATA1 OSs of the alternate cluster used reciprocally as background.

MEG

Overlapping OSs MEG/HPC-7

HPC7	MEG	No of occupied genes	All MEG-regulated genes			Non-primed	Primed	1	2	3	4	5	6	7	8	9
		MEG	U	D	N	U	U	U	D	U	D	U	D	N	N	N
		ERY	-	-	-	-	-	U	D	D	U	N	N	U	D	N
HPC7	MEG	7513	1103	1615	1154	166	203	380	898	134	93	171	121	181	173	280
ERG		6817	-1.1	.	.	-1.5	.	-1.1	.	.	-1.4	-1.1	.	.	.	.
Fli1		4211	1.1	.	.	.	1.3	.	.	1.6	-1.5	.	.	.	1.4	.
Gata2		1770	1.5	.	.	.	1.8	.	.	2.7	.	1.6	.	.	.	.
Lmo2		1964	1.6	.	.	1.6	1.9	1.3	.	2.7	.	1.5	.	.	1.5	-1.8
Lyl1		938	1.9	-1.3	.	1.8	2.2	1.5	.	4.1	.	1.8	.	.	2.2	.
PU1		5343	.	.	.	-1.6	1.1	.	.	1.2	-1.3	.	.	.	1.2	.
Runx1		1410	1.5	.	.	.	2.1	.	.	3.3	.	.	.	.	.	.
SCL		3082	.	.	.	.	1.6	.	.	1.7	.	.	.	.	1.3	.
	GATA1/TAL1	387	3.2	-4.2	.	.	4.9	2.2	-4.2	7.5	.	2.8	.	.	.	.
	GATA1	765	2.4	-1.8	.	.	3.1	1.8	-1.9	4.6	.	2.4	.	.	.	.
	TAL1	1149	2.1	-1.5	.	1.7	2.9	1.6	-1.4	3.9	.	2.1	.	.	.	.
	FLI1	1542	.	.	.	.	.	.	-1.3	1.7	.	.	.	.	.	.
	GATA2	863	1.5	.	.	.	2.0	.	.	2.7	.	.	.	.	.	.
ERG	GATA1/TAL1	286	3.1	-3.8	.	.	5.3	2.2	-3.8	8.0	.	.	.	.	.	.
ERG	GATA1	521	2.3	-1.8	.	.	3.5	.	-1.8	5.3	.	2.5	.	.	.	.
ERG	TAL1	514	2.7	-2.2	.	2.2	4.2	.	-2.1	5.9	.	2.3	.	.	2.3	.
ERG	FLI1	1462	.	.	.	.	.	.	.	1.6	-3.0	.	.	.	.	.
ERG	GATA2	449	1.7	.	.	.	3.0	.	.	3.7	.	.	.	.	.	.
Fli1	GATA1/TAL1	299	3.2	-3.8	.	.	4.7	2.0	-3.6	8.4	.	2.8	.	.	.	.
Fli1	GATA1	468	2.6	-2.1	.	.	3.9	.	-1.9	6.5	.	2.9	.	.	.	.
Fli1	TAL1	501	2.9	-2.3	.	2.3	4.5	.	-2.1	6.8	.	2.8	.	.	2.2	.
Fli1	FLI1	1012	.	.	.	.	.	.	.	2.2	-6.3	.	.	.	.	.
Fli1	GATA2	357	2.0	.	.	.	3.6	.	.	4.7	.	.	.	.	.	.
Gata2	GATA1/TAL1	217	3.2	-3.6	.	.	5.6	2.3	-2.9	8.8	.	.	.	.	.	.
Gata2	GATA1	344	2.7	-2.0	.	.	4.5	.	.	6.7	.	2.7	.	.	.	.
Gata2	TAL1	309	3.0	-2.7	.	.	5.0	2.0	-2.5	7.1	.	2.6	.	.	.	.
Gata2	FLI1	166	2.5	.	.	.	4.5	.	.	7.8	.	.	.	.	.	.
Gata2	GATA2	338	2.1	.	.	.	3.9	.	.	5.0	.	.	.	.	.	.
Lmo2	GATA1/TAL1	308	3.2	-4.1	.	.	5.5	2.3	-4.6	8.4	.	2.7	.	.	.	.
Lmo2	GATA1	401	2.9	-2.9	.	.	4.7	2.1	-3.0	7.4	.	2.8	.	.	.	.
Lmo2	TAL1	508	3.0	-2.3	.	2.4	4.7	2.0	-2.2	7.0	.	2.4	.	.	.	.
Lmo2	FLI1	250	2.5	.	.	.	4.4	.	.	6.5	.	.	.	.	.	.
Lmo2	GATA2	315	2.3	.	.	.	4.1	.	.	5.5	.	.	.	.	.	.
Lyl1	GATA1/TAL1	199	3.2	-4.3	.	.	4.8	2.4	-3.0	9.6	.	.	.	.	.	.
Lyl1	GATA1	245	3.0	-3.5	.	.	4.5	2.2	-2.7	8.5	.	2.9	.	.	.	.
Lyl1	TAL1	304	2.9	-2.4	.	.	4.1	2.2	.	7.7	.	.	.	.	.	.
Lyl1	FLI1	137	2.8	.	.	.	4.3	.	.	9.4	.	.	.	.	.	.
Lyl1	GATA2	215	2.5	.	.	.	4.0	.	.	6.0	.	.	.	.	.	.
PU1	GATA1/TAL1	116	2.8	-3.6	.	.	5.1	.	.	7.3	.	.	.	.	.	.
PU1	GATA1	260	1.9	.	.	.	3.0	.	.	4.5	.	.	.	.	.	.
PU1	TAL1	224	2.6	-2.0	.	.	4.1	.	.	5.8	.	.	.	.	.	.
PU1	FLI1	949	.	.	.	.	.	.	.	.	.	.	.	.	.	.
PU1	GATA2	307	.	.	.	.	.	.	.	2.9	.	.	.	.	.	.
Runx1	GATA1/TAL1	211	3.1	-3.8	.	.	5.4	2.2	-3.2	8.5	.	.	.	.	.	.
Runx1	GATA1	286	2.8	-2.8	.	.	4.5	.	-2.4	7.3	.	2.8	.	.	.	.
Runx1	TAL1	324	2.8	-2.0	.	.	4.6	.	.	6.7	.	.	.	.	.	.
Runx1	FLI1	246	2.1	.	.	.	3.5	.	.	6.6	.	.	.	.	.	.
Runx1	GATA2	244	2.4	.	.	.	4.1	.	.	5.7	.	.	.	.	.	.
SCL	GATA1/TAL1	279	3.1	-4.0	.	.	5.3	2.3	-4.2	8.0	.	2.7	.	.	.	.
SCL	GATA1	373	2.7	-2.7	.	.	4.4	2.1	-2.3	6.6	.	2.6	.	.	.	.
SCL	TAL1	453	2.8	-2.3	.	.	4.4	1.9	-2.2	6.3	.	2.4	.	.	.	.
SCL	FLI1	635	.	.	.	.	2.3	.	.	2.6	.	.	.	.	.	.
SCL	GATA2	293	2.1	.	.	.	3.8	.	.	5.2	.	.	.	.	.	.

Supplemental Figure 11. Enrichment of HPC-7 and MEG transcription factor OSs across developmental gene clusters. *U*, up-regulated. *D*, down-regulated. *N*, no change. The enrichment value is a ratio of the fraction of genes in a given cluster occupied by a given transcription factor, *versus* the fraction of occupied genes in the global expressed gene set of 7513 genes. All enrichments shown have *p*-values <0.001 by Fisher exact test.

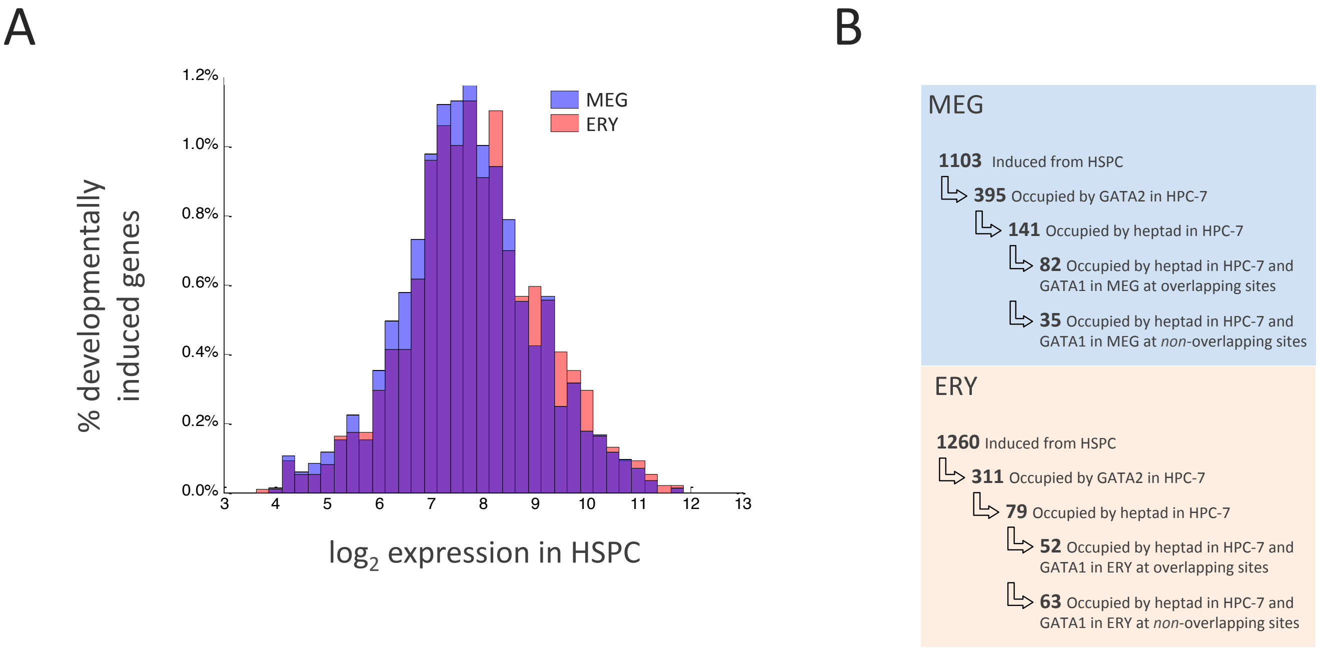

Supplemental Figure 12. Gene priming in ERY and MEG. *A*, Distribution of MEG and ERY-induced genes according to starting expression levels in HSPCs. Purple bars indicate areas of overlap between MEG and ERY. *B*, Numbers of GATA2 and heptad-occupied genes in MEG and ERY development.

ERY

			All ERY-regulated genes			Non-primed	Primed	1	2	3	4	5	6	7	8	9
		MEG	-	-	-	-	-	U	D	U	D	U	D	N	N	N
		ERY	U	D	N	U	U	U	D	D	U	N	N	U	D	N
HPC7	ERY	7513	1260	1865	1153	170	286	380	898	134	93	171	121	181	173	280
ERG		6817	-1.1	1.0	.	-2.0	.	-1.1	.	.	-1.4	-1.1	.	.	.	.
Fli1		4211	-1.2	1.2	.	-1.9	.	.	.	1.6	-1.5	.	.	.	1.4	.
Gata2		1770	.	1.3	.	.	.	.	.	2.7	.	1.6	.	.	.	.
Lmo2		1964	.	1.2	.	.	1.4	1.3	.	2.7	.	1.5	.	.	1.5	-1.8
Lyl1		938	.	1.4	.	.	.	1.5	.	4.1	.	1.8	.	.	2.2	.
PU1		5343	-1.1	1.1	.	-2.0	.	.	.	1.2	-1.3	.	.	.	1.2	.
Runx1		1410	.	1.4	.	.	.	.	.	3.3	.	.	.	.	.	.
SCL		3082	-1.2	.	.	-2.2	.	.	.	1.7	.	.	.	.	1.3	.
	GATA1/TAL1	1199	2.8	-2.9	.	2.2	3.6	2.8	-3.0	.	3.2	.	.	3.2	-2.3	.
	GATA1/TAL1	2411	1.9	-1.8	.	1.4	2.3	1.9	-2.0	.	1.9	.	.	2.1	-1.6	.
	TAL1	1352	2.7	-2.6	.	2.1	3.4	2.6	-2.9	.	2.9	.	.	2.9	-2.2	.
ERG	GATA1/TAL1	365	2.8	-2.2	.	.	4.0	3.3	-3.4	.	.	.	.	3.5	.	.
ERG	GATA1	1003	1.6	-1.5	.	.	2.1	1.9	-1.6	.	.	.	.	1.8	.	.
ERG	TAL1	437	2.7	-2.3	.	.	3.8	2.9	-3.7	.	.	.	.	3.4	.	.
Fli1	GATA1/TAL1	243	2.8	-2.2	.	.	4.1	3.7	-5.8	.	.	.	.	2.9	.	.
Fli1	GATA1	544	1.7	-1.6	.	.	2.5	2.2	-2.3	.	.	.	.	2.1	.	.
Fli1	TAL1	269	2.7	-2.2	.	.	3.9	3.4	-6.4	.	.	.	.	2.9	.	.
Gata2	GATA1/TAL1	227	2.6	-2.0	.	.	3.8	3.6	-3.0	.	.	.	.	2.9	.	.
Gata2	GATA1	494	1.8	-1.5	.	.	2.4	2.5	-1.9	.	.	.	.	.	.	.
Gata2	TAL1	243	2.6	-2.0	.	.	3.7	3.3	-3.2	.	.	.	.	2.9	.	.
Lmo2	GATA1/TAL1	368	3.1	-2.3	.	.	4.1	4.4	-3.1	.	.	.	.	3.3	.	.
Lmo2	GATA1	463	2.7	-1.8	.	.	3.6	3.8	-2.5	.	.	.	.	3.0	.	.
Lmo2	TAL1	394	3.0	-2.2	.	2.2	3.9	4.2	-3.1	.	.	.	.	3.1	.	.
Lyl1	GATA1/TAL1	162	2.8	-2.1	.	.	4.4	4.0	-3.9	.	.	.	.	3.1	.	.
Lyl1	GATA1	203	2.6	.	.	.	4.0	3.7	-3.5	.	.	.	.	3.3	.	.
Lyl1	TAL1	173	2.6	-2.1	.	.	4.1	3.8	-4.1	.	.	.	.	.	.	.
PU1	GATA1/TAL1	153	2.5	.	.	.	3.6	2.8	.	.	.	.	.	4.1	.	.
PU1	GATA1	533	1.6	-1.5	.	.	2.0	1.8	-1.9	.	.	.	.	2.0	.	.
PU1	TAL1	198	2.4	.	.	.	3.3	2.4	.	.	.	.	.	3.6	.	.
Runx1	GATA1/TAL1	178	2.9	-2.0	.	.	4.4	3.8	.	.	.	.	.	.	.	.
Runx1	GATA1	242	2.5	-1.9	.	.	3.8	3.3	-2.4	.	.	.	.	.	.	.
Runx1	TAL1	195	2.6	.	.	.	4.0	3.4	.	.	.	.	.	.	.	.
SCL	GATA1/TAL1	291	2.8	-2.0	.	.	4.3	3.7	-3.2	.	.	.	.	3.6	.	.
SCL	GATA1	529	1.9	-1.8	.	.	2.7	2.4	-2.0	.	.	.	.	2.3	.	.
SCL	TAL1	323	2.7	-2.1	.	.	4.1	3.5	-3.5	.	.	.	.	3.5	.	.

Supplemental Figure 13. Enrichment of HPC-7 and ERY transcription factor OSs across developmental gene clusters. *U*, up-regulated. *D*, down-regulated. *N*, no change. The enrichment value is a ratio of the fraction of genes in a given cluster occupied by a given transcription factor, *versus* the fraction of occupied genes in the global expressed gene set of 7513 genes. All enrichments shown have *p*-values <0.001 by Fisher exact test.

MEG

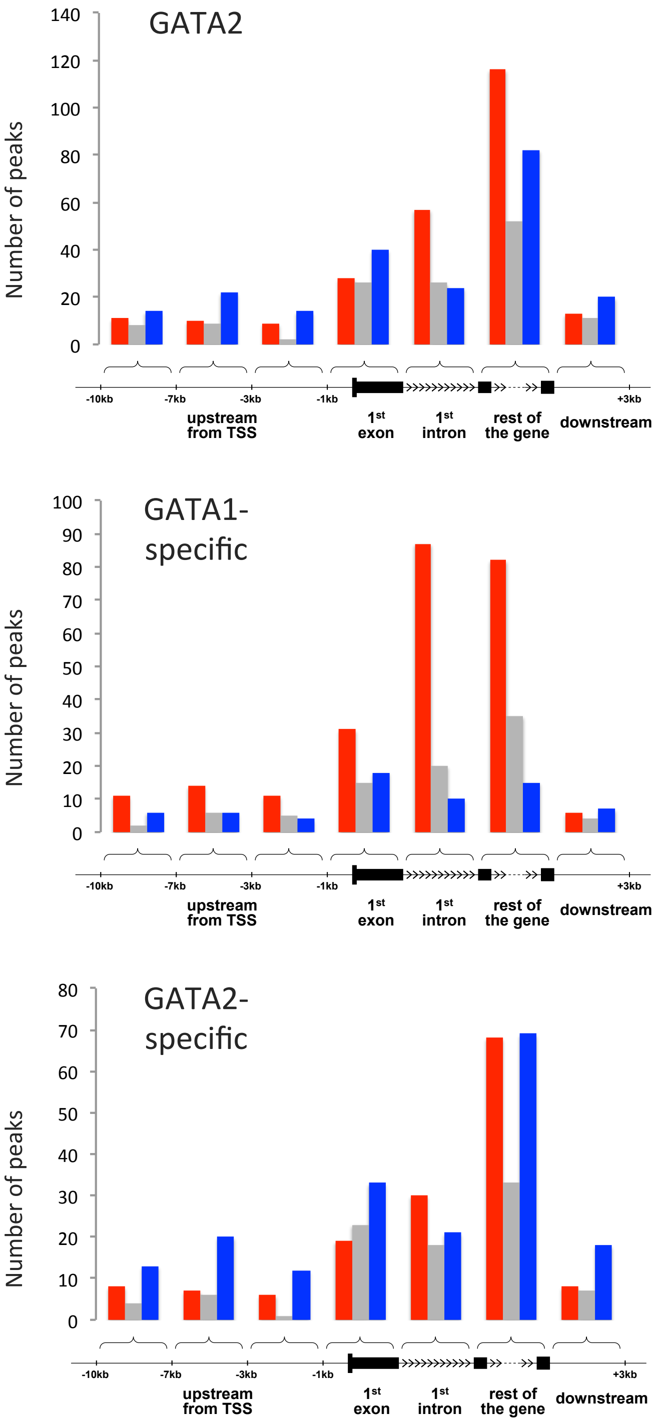

Supplemental Figure 14. Distribution of transcription factor OSs across gene structure and its correlation with the developmental gene expression response in MEG. Distribution of GATA2 (all), GATA2-specific and GATA1-specific OSs across the gene structure, shown as the absolute number of peaks called in a given gene segment, in up-regulated (red), down-regulated (blue) and non-responsive (grey) gene categories. Introns are represented by thin lines with arrows marking the direction of transcription.

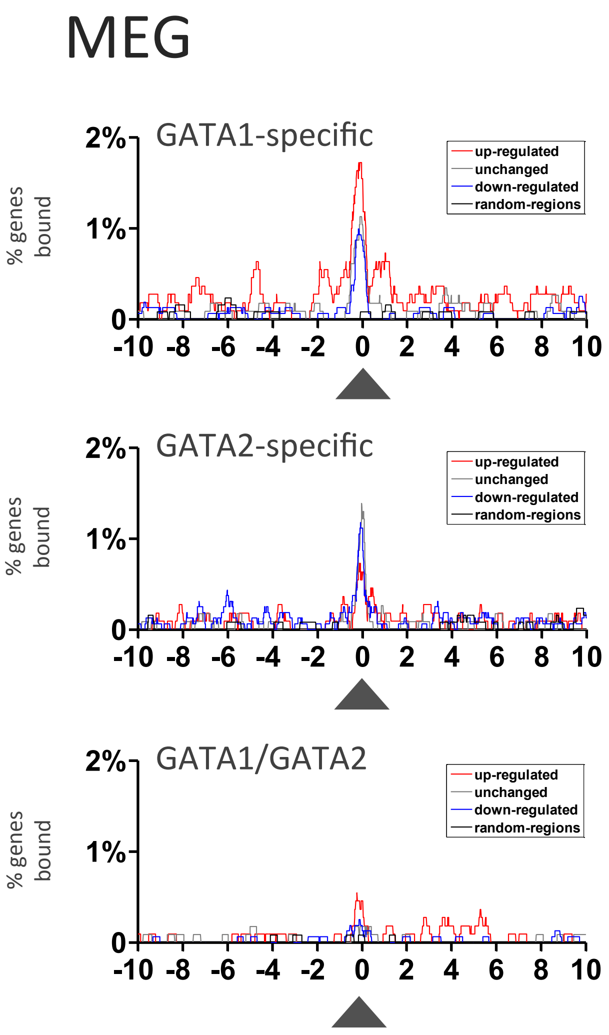

Supplemental Figure 15. Distribution of GATA OSs sites relative to TSS (indicated by black arrows) in up-regulated, down-regulated and non-responsive gene categories in MEG. Horizontal axis, Kb from upstream or downstream from TSS (arrow). Vertical axis, % genes in a given response category with at least one transcription factor binding peak.
