# Supplementary material for: Divergent functions of hematopoietic transcription factors in lineage priming and differentiation during erythro-megakaryopoiesis
Source: Genome Res. 2014 Dec;24(12):1932–44. doi: 10.1101/gr.164178.113 (PMC4248311; doi:10.1101/gr.164178.113)
Supplement: Supplemental Material [file supp_gr.164178.113_Supplemental_Methods.pdf]

Divergent functions of hematopoietic transcription factors in lineage priming and differentiation during erythro-megakaryopoiesis

SUPPLEMENTAL METHODS

Maxim Pimkin[1,6,8], Andrew V. Kossenkov[2,6], Tejaswini Mishra[3,4], Christapher S. Morrissey[3,4], Weisheng Wu[3,4], Cheryl A. Keller[3,4], Gerd A. Blobel[1,5], Dongwon Lee[7], Michael A. Beer[7], Ross C. Hardison[3,4] and Mitchell J. Weiss[1]

[1]Division of Hematology, The Children's Hospital of Philadelphia, PA
[2]Center for Systems and Computational Biology, The Wistar Institute, Philadelphia, PA
[3]Center for Comparative Genomics and Bioinformatics, Pennsylvania State University, University Park, PA
[4]Departments of Biochemistry and Molecular Biology, Pennsylvania State University, University Park, PA
[5]University of Pennsylvania Perelman School of Medicine, Philadelphia, PA
[6]These authors contributed equally to this work
[7]McKusick-Nathans Institute of Genetic Medicine and Department of Biomedical Engineering, Johns Hopkins University School of Medicine, Baltimore, MD
[8]Pediatric Residency Program, Department of Pediatrics, Children's Hospital of Pittsburgh of UPMC, University of Pittsburgh School of Medicine, Pittsburgh, PA

Correspondence:

Mitchell J. Weiss, The Children's Hospital of Philadelphia, 316ARC, 3615 Civic Center Blvd, Philadelphia, PA 19104; weissmi@email.chop.edu

Ross C. Hardison, Pennsylvania State University, 304 Wartik Laboratory, University Park, PA 16802; rch8@psu.edu

Cell culture and purification

Fetal livers were harvested from E14.5 pregnant mice and used as a source of all cell populations in this study. For purification of primary mouse fetal erythroblasts, the fetal liver cell suspension was labeled with a PE-conjugated anti-Ter119 antibody. EasySep PE Selection Kit (#18554) was then used to purify Ter119-positive, PE-labeled erythroblasts.

For MEG production, we used the mouse CD117 (cKit) EasySep selection kit (#18757) to purify cKit-positive hematopoietic progenitors in full accordance with the manufacturer's recommendations. The cKit-positive cells were incubated for 4-6 days in a cell culture medium containing mouse stem cell factor (mSCF) and thrombopoietin (TPO) to promote expansion of MEG progenitors. The cells were then washed and incubated for 5 more days in a cell culture medium in the presence of TPO alone to promote terminal MEG differentiation. EasySep PE Selection Kit (#18554) was used to purify CD14-positive MEG labeled with a PE-conjugated anti-CD41 antibody.

For purification of HSPC, the E14.5 fetal liver cell suspension was enriched for hematopoietic progenitors using EasySep Hematopoietic Progenitor Cell Enrichment Kit (#19756). Briefly, fetal liver cell suspension was stained with biotinylated antibodies against lineage antigens (CD5, CD11b, CD19, CD45R, Ly-6G/C, Ter119, CD71), followed by removal of lineage-positive cells using a magnetic bead protocol. The resulting population was stained with an APC-conjugated anti-Sca-1 antibody, and the Sca1-positive hematopoietic progenitors (HSPC) were purified by FACS sorting. Non-viable cells and cellular debris were excluded based on forward and side scatter characteristics.

Transcriptome analysis

mRNA was extracted with phenol-chlorophorm and further purified using Qiagen RNeasy purification kit. GeneChip® Mouse Gene 1.0 ST Arrays were used to interrogate genome-wide mRNA expression of HSPC, MEG and ERY cells using 4 biological replicates for each cell type. The raw hybridization data were corrected for background, quantile-normalized and log2-transformed using RMA (Irizarry et al., 2003). Probesets were then filtered to keep only those that had expression values more than 8.69 for at least 1 sample. This cutoff value was calculated as 95th percentile of the

distribution of expression values for negative control probes across all samples. The filtering resulted in 8684 probesets. All probesets were then tested for differential expression between each pair of groups and corrected for multiple testing by the Benjamini-Hochberg approach. Then, we removed 1171 probesets to keep only those that represented unique transcripts, preferentially discarding redundant probesets with the smallest significant fold change between the three cell types. The filtering resulted in a final list of 7513 probesets significantly expressed in at least one sample of at least one cell type. We used the HSPC transcriptome as a reference point to define the developmental changes in gene expression during mono-lineage differentiation. For MEG *vs.* HSPC and ERY *vs.* HSPC comparisons, genes that significantly (FDR < 5%) changed more than 2-fold relative to the expression level in HSPC were considered to be developmentally up- or down-regulated. Genes whose expression was altered insignificantly (nominal p > 0.05) or less than 1.2-fold were considered to be unchanged (**Figure 2A**). Genes that changed between 1.2 and 2-fold were considered indeterminable and not examined further in this study. Differential expression of selected signature genes was confirmed by TaqMan RT-PCR (**Supplemental Figure7**).

A heatmap for a list of genes was composed using hierarchical clustering with Spearman correlation distance to cluster genes (**Supplemental Figure 7A**), or using predefined gene clustering with genes separated into 9 groups according to the bilineage MEG/ERY pattern of expression relative to that of HPSC (**Figure 3A**). Heatmap color intensities were proportional to the value calculated as a ratio between the gene expression in a single sample and the geometric mean expression of the gene across a set of samples.

Chromatin immunoprecipitation and massive parallel DNA sequencing

For each transcription factor-cell type combination, ChIP-seq was performed on two to four biological replicates. We used the approach of irreproducible discovery rate at a threshold of 0.02 to determine the number *n* of reproducible peaks in the replicate datasets (Landt et al., 2012; Li et al., 2011). Peaks were called on the combined reads from all replicates and the top *n* peaks were taken as the set of high confidence peaks. This is a very conservative method for thresholding, and we also generated a larger set of quality peaks, called the reduced stringency peaks, by applying a threshold based on the *p*-value of the least significant peak in the top 90% of reproducible peaks (the threshold *p*-values ranged from 10^{-80} to 10^{-160}; a detailed description is presented below)

The high stringency set of peaks was used for all analyses, and for cases in which the smaller number of peaks could affect the interpretation, the analysis was repeated for the larger set of reduced stringency peaks. For all samples except GATA2 in megakaryocytes two to four biological replicates were performed.

Antibodies used

(1) TAL1: Santa Cruz Biotechnology, sc-12984 (2) GATA1: Santa Cruz Biotechnology, sc-265 (3) FLI1: Santa Cruz Biotechnology, sc-356 (4) GATA2: Santa Cruz Biotechnology, sc-9008 (5) H3K4me1: Abcam, ab8895 (6) H3K4me3: Millipore catalog number 07-473 (7) H3K27me3: Millipore catalog number 07-449

ChIP

ChIP assay was performed as previously described (Welch et al., 2004). Briefly, 75 million cells in PBS were crosslinked for 10 mins by adding formaldehyde at a final concentration of 0.4% and glycine was added at a final concentration of 125mM to quench cross-linking. For megakaryocytes, which are multiploid (6–64N), ~12 million cells in PBS were used for each ChIP assay. Cells were then lysed followed by nuclear lysis and sonication to shear the cross-linked chromatin. A Misonix S-4000 sonicator was used to shear samples in 8 repeats of 30 cycles of 1 sec. on, 1 sec. off sonication at output power 30. Fragments in the size range of 200-400bp were obtained. Sonicated chromatin was pre-cleared overnight at 4°C with 20 µg appropriate non-immune sera (IgG) on protein G agarose beads. 20 µg of the appropriate ChIP antibody were also pre-bound to protein G agarose beads overnight at 4°C. For binding, pre-cleared chromatin was added to the antibody:bead complex and incubated with rotation at 4°C for 2 – 4 hours. 200 µL of pre-cleared chromatin was saved for use as input. After binding, the beads were washed with wash buffers, high-salt buffer and TE. DNA:protein complexes were eluted from beads into an elution buffer (1% SDS, 100mM $NaHCO_3$). After adding 5M NaCl to ChIP and input samples, they were incubated overnight at 65 °C with 1µg RNase A. To digest protein, each sample was treated with 60 µg Proteinase K for 2 hours at 45 °C and immunoprecipitated DNA was finally extracted using the Qiagen PCR Purification Kit.

Illumina Library Preparation

All samples including input were processed for library construction for Illumina sequencing using Illumina's ChIP-seq Sample Preparation Kit. DNA fragments were repaired to generate blunt ends and a single 'A' nucleotide was added to each end. Double-stranded Illumina adaptors were ligated to the fragments. Ligation products were amplified by 18 cycles of PCR, and the DNA between 250-350 bp was gel purified. Completed libraries were quantified with Quant-iT dsDNA HS Assay Kit. The DNA library was sequenced on the Illumina Genome Analyzer II sequencing system, and more recently on the HiSeq. Cluster generation, linearization, blocking and sequencing primer reagents were provided in the Illumina Cluster Amplification kits.

Data processing, peak calls, assessment of quality and reproducibility of ChIP-seq data

Data processing and peak calling

Raw ChIP-seq reads were first groomed using FASTQ Groomer on Galaxy (Blankenberg et al., 2010; Giardine et al., 2005; Goecks et al., 2010). This program verifies that each base call has a corresponding quality value, and that the quality value is in the Sanger Phred+33 format. Groomed reads were then mapped to mouse mm9 genome using Bowtie (Langmead et al., 2009) using the parameters –m = -1 (no limit), -k = 1, and –best, thus allowing reads to map to multiple locations, but reporting only the single, best alignment. This option was chosen to allow reads to map in duplicated regions such as those containing the Hba genes. The mapped reads for a transcription factor as well as input reads for the appropriate cell line are then passed to MACS (Zhang et al., 2008) for peak calling using an mfold of 12, p-value threshold of 1e-05 and bw set to half the ChIP DNA fragment length as measured on an Agilent Bioanalyzer. To insure that all of the experiments were processed consistently, all of the above steps were performed as part of a Galaxy workflow, which can be found at *https://main.g2.bx.psu.edu/u/csm165/w/prototypehardisonchip-seqworkflow---mm9-canonical-male*

Quality assessment

Another method for measuring the quality of a ChIP-seq experiment, Cross-Correlation, was developed by Anshul Kundaje et al (Kundaje et al., submitted) (Landt et al., 2012). This analysis uses the cross-correlation profile of the mapped reads from a given

experiment to highlight the extent of enrichment achieved by immunoprecipitation. A well enriched sample that has been sequenced to sufficient depth should show a peak in the cross-correlation profile at a distance equivalent to the average fragment length of the sample, as positive and negative strand reads pile up on either side of the position occupied by a transcription factor. A sample that is poorly enriched will show a peak at approximately the length of the reads generated by the sequencer. A ratio of the heights of the peaks at these two positions, the rPhc, can be used as metric for identifying the extent of enrichment an experiment achieved. Results of this assessment are summarized in the table below. Larger values of rPhc indicate higher quality experiments. A summary of the datasets, including number of mapped reads and quality assessment metrics is presented in the table below (**Supplemental Methods Table 1**).

Sample	Platform	Mapped reads	rPhc	Qtag
ERY TAL1/SCL				
Replicate1	GAIIx + HiSeq	126,692,402	1.49	1
Replicate2	HiSeq	97,409,304	1.36	1
ERY GATA1				
Replicate1	GAIIx	33,444,157	1.54	2
Replicate2	HiSeq	77,520,334	1.05	1
MEG TAL1				
Replicate1	GAIIx	14,026,574	2.05	2
Replicate2	HiSeq	105,068,277	1.00	1
Replicate3	HiSeq	125,755,601	1.21	1
Replicate4	HiSeq	55,085,957	0.91	0
MEG GATA1				
Replicate1	GAIIx	29,561,738	1.10	1
Replicate2	HiSeq	82,769,534	0.84	0
Replicate3	HiSeq	49,859,498	0.60	0
MEG GATA2				
Replicate1	HiSeq	97,251,129	0.96	0
MEG FLI1				
Replicate1	HiSeq	85,007,549	0.81	0
Replicate2	HiSeq	108,383,696	0.92	0

Supplemental Methods Table 1. A summary of ChIP-Seq experiments and data analysis.

Consistency analysis for measuring reproducibility between replicates

For all ChIP-seq experiments other than GATA2 in megakaryocytes, a minimum of two biological replicates were sequenced. To assess the level of reproducibility between our replicates, we used a recently published method, called irreproducible discovery rate analysis (IDR), which was designed for analysis of consistency between replicates in high-throughput experiments (Li et al., 2011). This method not only reveals the degree of

reproducibility between replicates, but also provides an objective criterion called the irreproducible discovery rate (IDR), to control or report the level of irreproducibility, in a fashion similar to the FDR. This method has been used extensively by the ENCODE and modENCODE Consortia to objectively identify high-confidence peaks from datasets generated by several labs (Mouse ENCODE Consortium et al., 2012).

We performed IDR analysis as described in the ENCODE standards manuscript, with a few modifications (Landt et al., 2012). The method is described in detail at https://sites.google.com/site/anshulkundaje/projects/idr, ("IDR on original replicates"). We used MACS to call peaks with a relaxed p-value threshold of 0.05 for individual replicates as well as the pooled data using the following workflows, respectively:

https://main.g2.bx.psu.edu/u/csm165/w/adjustablepvaluehardisonidrpeakcalling

https://main.g2.bx.psu.edu/u/csm165/w/adjustablepooledhardisonchip-seqworkflow

This resulted in a minimum of 100,000 peaks per replicate, and was expected to contain many false positives. The peaks for each pair of replicates were then run through the IDR pipeline using the p-values as a ranking measure ('ranking.measure' = p.value), with overlapping peaks defined as peaks with >=1bp overlap ('min.overlap.ratio' =0). As recommended on the above IDR webpage, IDR of 0.02 was used as a threshold to identify the number N of reproducible peaks from the pairs of replicates. For samples with more than two replicates, we performed pairwise consistency analysis for all the possible pairs of replicates the reproducible peaks from each pairwise comparison were concatenated and merged to give a set of N non-overlapping peaks. We obtained a final set of high-confidence peaks for each factor by selecting the top N peaks from the set of pooled peaks ranked by fold-enrichment. This is a very stringent set of peaks. We also obtained a larger set of less conservative set of peaks, called the confident peaks by selecting peaks from the pooled set whose p-value was more significant than TF-90, where TF-90 was defined as -10log10(p-value) of the least significant reproducible peak out of the top 90th percentile of reproducible peaks. For both sets, ChIP-seq peaks overlapping blacklisted regions (see below) were first removed from the set of pooled

peaks prior to selection of final peaks. A summary of the IDR results can found in the **Supplemental Methods Table 2** below.

<table>
<tr><th>Sample</th><th>Num peaks IDR <= 0.02</th><th>Num of high-confidence peaks, N)</th><th>TF-90</th><th>Num of confident peaks (N90)</th></tr>
<tr><td>TAL1 Megs</td><td></td><td></td><td></td><td></td></tr>
<tr><td>R1-R2</td><td>1475</td><td rowspan="6">3505</td><td rowspan="6">81.06</td><td rowspan="6">4617</td></tr>
<tr><td>R1-R3</td><td>2475</td></tr>
<tr><td>R1-R4</td><td>1116</td></tr>
<tr><td>R2-R3</td><td>2316</td></tr>
<tr><td>R2-R4</td><td>1132</td></tr>
<tr><td>R3-R4</td><td>906</td></tr>
<tr><td>GATA1 Megs</td><td></td><td></td><td></td><td></td></tr>
<tr><td>R1-R2</td><td>655</td><td rowspan="3">1727</td><td rowspan="3">59.47</td><td rowspan="3">7421</td></tr>
<tr><td>R2-R3</td><td>630</td></tr>
<tr><td>R1-R3</td><td>1536</td></tr>
<tr><td>FLI1 Megs</td><td></td><td></td><td></td><td></td></tr>
<tr><td>R1-R2</td><td>2001</td><td>2001</td><td>81.49</td><td>2763</td></tr>
<tr><td>TAL1 TER119+</td><td></td><td></td><td></td><td></td></tr>
<tr><td>R1-R2</td><td>3086</td><td>3086</td><td>157.09</td><td>3211</td></tr>
<tr><td>GATA1 TER119+</td><td></td><td></td><td></td><td></td></tr>
<tr><td>R1-R2</td><td>5767</td><td>5767</td><td>60.37</td><td>6192</td></tr>
</table>

Supplemental Methods Table 2. A summary of the IDR data

Blacklist

As a final quality filter, we identified a set of genomic locations characterised by very high signal in our input or control tracks. We compiled a set of these locations by using MACS to identify peaks in the input tracks. To this list, we added additional locations, which showed elevated input signal over large areas (> 10,000 bp). We have identified the combination of these two groups of segments as a blacklist.

Data analysis

Peak intersections

Overlap between two peak regions was called between two transcription factors in the same lineage or between two lineages for the same transcription factors if the coordinates overlapped by at least 1 base pair. A gene was considered to be occupied in both lineages or by both transcription factors in two different ways: (1) if it had at least one peak overlapping the gene region where the "gene" includes 10 kb upstream of the TSS and 3 kb downstream of the polyA signal in both compared instances (2) if it had overlapped peak regions overlapping the gene region.

Motif enrichment

Enrichment of a motif was performed de novo, using HOMER algorithm (Heinz et al., 2010), testing for 8 to 16 base pair motif sequences. For enrichments among a set of peaks, a 200 bp window around the center of the peak was used as the target sequence. For motif enrichments within a set of genes we used the region from 2000 bp upstream to 500 bp downstream from the gene TSS. The canonical binding site motifs are enriched in the OSs from both lineages, and thus are not returned in the discriminative analysis.

Methylation profiles

The methylation profile for a set of transcription profile peaks was plotted for the region spanning -10 kb to +10 kb from the peak center. We used positive strands with a 200 bp window resolution. For heatmap generation, gray-scaled intensities were proportional to the significance (-log10 scale) of the methylation signal as compared to control with a maximum significance set at log10(P)=10 (black) and minimum log10(P)=0 (white). Individual rows were sorted by the significance of transcription factor binding peak signal with the methylation profile.

Enrichment of transcription factor occupancy and histone methylation marks

The Fisher exact test was used to test if occupancy by a transcription factor or the presence of a methylation mark is over- or under-represented in a gene group (G) compared to all considered genes (A). For n being the number of genes occupied by a transcription factor in a gene set and N being a total number of genes in a set, the

following values were used for Fisher exact test: Gn, GN-Gn, An, AN-An. The enrichment value was calculated as a ratio: Enrichment = Gn/GN/ (An/AN). Enrichments of less than 1 (under-representation of a transcription factor binding or a histone methylation mark) were converted to -1/enrichment values. Statistical significance was defined at $p<0.001$.

Ingenuity

Pathway, function and upstream regulator enrichment analysis was carried out with the Ingenuity Pathways Analysis software (http://www.ingenuity.com/) using Ingenuity Core Analysis (IPA 8.0, Ingenuity® Systems), with Benjamini-Hochberg correction for multiple testing and using $p<0.05$ as a significance threshold.

REFERENCES

Blankenberg, D., Kuster, Von, G., Coraor, N., Ananda, G., Lazarus, R., Mangan, M., Nekrutenko, A., and Taylor, J. (2010). Galaxy: a web-based genome analysis tool for experimentalists. Curr Protoc Mol Biol *Chapter 19*, Unit19.10.1–Unit19.10.21.

Giardine, B., Riemer, C., Hardison, R.C., Burhans, R., Elnitski, L., Shah, P., Zhang, Y., Blankenberg, D., Albert, I., Taylor, J., et al. (2005). Galaxy: a platform for interactive large-scale genome analysis. Genome Res *15*, 1451–1455.

Goecks, J., Nekrutenko, A., Taylor, J., Galaxy Team (2010). Galaxy: a comprehensive approach for supporting accessible, reproducible, and transparent computational research in the life sciences. Genome Biol *11*, R86.

Heinz, S., Benner, C., Spann, N., Bertolino, E., Lin, Y.C., Laslo, P., Cheng, J.X., Murre, C., Singh, H., and Glass, C.K. (2010). Simple combinations of lineage-determining transcription factors prime cis-regulatory elements required for macrophage and B cell identities. Mol Cell *38*, 576–589.

Irizarry, R.A., Bolstad, B.M., Collin, F., Cope, L.M., Hobbs, B., and Speed, T.P. (2003). Summaries of Affymetrix GeneChip probe level data. Nucleic Acids Res *31*, e15.

Landt, S.G., Marinov, G.K., Kundaje, A., Kheradpour, P., Pauli, F., Batzoglou, S., Bernstein, B.E., Bickel, P., Brown, J.B., Cayting, P., et al. (2012). ChIP-seq guidelines and practices of the ENCODE and modENCODE consortia. Genome Res *22*, 1813–1831.

Langmead, B., Trapnell, C., Pop, M., and Salzberg, S.L. (2009). Ultrafast and memory-efficient alignment of short DNA sequences to the human genome. Genome Biol *10*, R25.

Li, Q., Brown, J.B., Huang, H., and Bickel, P.J. (2011). Measuring reproducibility of high-

throughput experiments. Ann. Appl. Stat. *5*, 1752–1779.

Mouse ENCODE Consortium, Stamatoyannopoulos, J.A., Snyder, M., Hardison, R., Ren, B., Gingeras, T., Gilbert, D.M., Groudine, M., Bender, M., Kaul, R., et al. (2012). An encyclopedia of mouse DNA elements (Mouse ENCODE). Genome Biol *13*, 418.

Welch, J.J., Watts, J.A., Vakoc, C.R., Yao, Y., Wang, H., Hardison, R.C., Blobel, G.A., Chodosh, L.A., and Weiss, M.J. (2004). Global regulation of erythroid gene expression by transcription factor GATA-1. Blood *104*, 3136–3147.

Zhang, Y., Liu, T., Meyer, C.A., Eeckhoute, J., Johnson, D.S., Bernstein, B.E., Nusbaum, C., Myers, R.M., Brown, M., Li, W., et al. (2008). Model-based analysis of ChIP-Seq (MACS). Genome Biol *9*, R137.
