# Supplementary material for: Divergent functions of hematopoietic transcription factors in lineage priming and differentiation during erythro-megakaryopoiesis
Source: Genome Res. 2014 Dec;24(12):1932–44. doi: 10.1101/gr.164178.113 (PMC4248311; doi:10.1101/gr.164178.113)
Supplement: Supplemental Material [file supp_gr.164178.113_Supplemental_Tables.pdf]

Supplementary Table 1. Developmental priming of MEG genes by the GATA2/LYL1/ TAL1/FLI1/ERG/RUNX1/LMO2 transcription factor heptad. The table shows developmentally induced MEG genes that are occupied by the heptad in HPC-7 cells (Wilson et al, 2010) and by GATA1 in maturing MEG cells, respectively, at overlapping OSs. The directionality of the developmental response in the alternate lineage is also shown along with the fold change of gene expression. Blank values indicate indeterminable response (fold change between 1.2 and 2, or FDR > 0.05). Selected MEG-specific genes are **bolded**. Common MEG-ERY primed genes are *italicized*.

Accession No	Description	Gene symbol	Expression in HSPC	Expression change in MEG, fold	Expression change in ERY, fold	Response in MEG	Response in ERY	Bound by heptad in HPC-7	Bound by heptad in HPC-7 and GATA1 in MEG at overlapping site	Bound by heptad in HPC-7 and TAL1 in MEG at overlapping site
NM_175367	*stonin-2*	*Ston2*	7.86	8.49	3.66	up	up	yes	no	no
NM_018784	*type 2 lactosamine alpha-2,3-sialyltransferase*	*St3gal6*	7.13	8.39	6.24	up	up	yes	yes	yes
NM_010433	*homeodomain-interacting protein kinase 2 isoform*	*Hipk2*	9.36	3.66	6.74	up	up	yes	no	no
NM_133167	*beta-parvin*	*Parvb*	7.86	20.29	3.75	up	up	yes	no	no
NM_010587	*intersectin-1 isoform 1*	*Itsn1*	6.42	3.73	7.12	up	up	yes	yes	yes
NM_138953	*RNA polymerase II elongation factor ELL2*	*Ell2*	6.87	6.52	28.53	up	up	yes	no	yes
NM_028759	*DDB1- and CUL4-associated factor 6*	*Dcaf6*	7.28	2.91	5.97	up	up	yes	no	no
NM_013862	*rab GTPase-activating protein 1-like isoform a*	*Rabgap1l*	6.52	6.33	5.93	up	up	yes	no	no
NM_010026	*arf-GAP with SH3 domain, ANK repeat and PH*	*Asap1*	8.77	4.49	4.68	up	up	yes	no	no
NM_001081232	*hypothetical protein LOC320661*	*D5Ertd579e*	5.55	4.52	12.84	up	up	yes	no	no
NM_001081308	*serine/threonine-protein kinase TAO3*	*Taok3*	8.29	3.91	2.43	up	up	yes	no	yes
NM_026148	*LIM and senescent cell antigen-like-containing*	*Lims1*	8.49	9.02	2.87	up	up	yes	yes	yes
NM_023605	*F-box only protein 9 isoform 1*	*Fbxo9*	7.63	3.15	5.66	up	up	yes	yes	yes
NM_172473	*E3 ubiquitin-protein ligase HACE1*	*Hace1*	7.09	3.13	10.74	up	up	yes	no	no
NM_145541	*ras-related protein Rap-1A precursor*	*Rap1a*	8.89	8.22	4.93	up	up	yes	yes	yes
NM_026735	*mps one binder kinase activator-like 1A*	*Mobkl1a*	7.84	7.85	3.76	up	up	yes	no	no
NM_026921	*iron-sulfur cluster assembly 1 homolog,*	*Isca1*	8.11	3.07	7.84	up	up	yes	yes	yes
NM_146025	*sterile alpha motif domain-containing protein 14*	*Samd14*	8.49	4.67	5.12	up	up	yes	no	no

Accession No	Description	Gene symbol	Expression in HSPC	Expression change in MEG, fold	Expression change in ERY, fold	Response in MEG	Response in ERY	Bound by heptad in HPC-7	Bound by heptad in HPC-7 and GATA1 in MEG at overlapping site	Bound by heptad in HPC-7 and TAL1 in MEG at overlapping site
NM_001164557	*PDZK1-interacting protein 1 isoform 1*	*Pdzk1ip1*	7.27	4.05	4.38	up	up	yes	no	yes
NM_153538	*terminal uridylyltransferase 7*	*Zcchc6*	7.87	3.23	8.58	up	up	yes	yes	yes
NM_145486	*E3 ubiquitin-protein ligase MARCH2*	*40604*	9.18	3.27	6.85	up	up	yes	yes	yes
NM_028011	*TOM1-like protein 1*	*Tom1l1*	7.69	6.55	7.48	up	up	yes	yes	yes
NM_026004	*cytosolic 5'-nucleotidase 3*	*Nt5c3*	7.03	9.18	11.47	up	up	yes	yes	no
NM_001080977	*round spermatid basic protein 1-like*	*Rsbn1l*	7.65	7.35	3.84	up	up	yes	no	no
NM_021461	*MAP kinase-interacting serine/threonine-protein*	*Mknk1*	8.99	5.74	2.31	up	up	yes	yes	yes
NM_016920	*V-type proton ATPase 116 kDa subunit a isoform*	*Atp6v0a1*	8.60	6.08	7.31	up	up	yes	yes	yes
NM_011158	*cAMP-dependent protein kinase type II-beta*	*Prkar2b*	8.66	12.05	5.61	up	up	yes	yes	yes
NM_009177	*CMP-N-acetylneuraminate-beta-galactosamide-*	*St3gal1*	9.68	2.77	2.35	up	up	yes	yes	yes
NM_001168525	*phosphatidylcholine:ceramide*	*Sgms1*	8.04	3.87	3.11	up	up	yes	yes	yes
NM_173755	*ubiquitin-conjugating enzyme E2 O*	*Ube2o*	8.52	4.85	10.65	up	up	yes	no	yes
NM_011170	*major prion protein precursor*	*Prnp*	5.58	3.00	8.21	up	up	yes	no	no
NM_009179	*CMP-N-acetylneuraminate-beta-galactosamide-*	*St3gal2*	7.79	2.74	3.97	up	up	yes	no	yes
NM_008207	*histocompatibility 2, T region locus 24*	*H2-T24*	6.79	6.76	7.21	up	up	yes	no	yes
NM_177857	*DENN domain-containing protein 2C*	*Dennd2c*	6.27	12.94	14.80	up	up	yes	yes	yes
NM_023738	*ubiquitin-like modifier-activating enzyme 7*	*Uba7*	7.63	4.14	2.92	up	up	yes	yes	yes
NM_001033550	*leucine-rich repeat-containing protein 8B*	*Lrrc8b*	9.15	3.42	3.47	up	up	yes	yes	yes
NM_001033488	*major facilitator superfamily domain-containing*	*Mfsd2b*	9.27	4.22	3.08	up	up	yes	yes	yes
NM_008982	receptor-type tyrosine-protein phosphatase eta	Ptprj	8.32	12.25	-1.00	up	nch	yes	no	no
NM_139272	polypeptide N-acetylgalactosaminyltransferase 2	Galnt2	9.14	3.14	-1.13	up	nch	yes	no	no
NM_207708	synaptogyrin-1 isoform 1a	Syngr1	8.09	3.46	1.16	up	nch	yes	no	yes
NM_027238	tetratricopeptide repeat protein 39B	Ttc39b	5.34	18.17	-1.08	up	nch	yes	yes	yes
NM_134250	hepatitis A virus cellular receptor 2 homolog	Havcr2	5.55	9.56	1.15	up	nch	yes	yes	yes
NM_029166	UHRF1-binding protein 1-like	Uhrf1bp1l	8.14	6.10	1.15	up	nch	yes	yes	yes

Accession No	Description	Gene symbol	Expression in HSPC	Expression change in MEG, fold	Expression change in ERY, fold	Response in MEG	Response in ERY	Bound by heptad in HPC-7	Bound by heptad in HPC-7 and GATA1 in MEG at overlapping site	Bound by heptad in HPC-7 and TAL1 in MEG at overlapping site
NM_029153	secretory carrier-associated membrane protein 1	Scamp1	8.31	3.70	-1.07	up	nch	yes	yes	no
NM_009624	adenylate cyclase type 9	Adcy9	7.00	6.63	-1.09	up	nch	yes	yes	yes
NM_007858	protein diaphanous homolog 1	Diap1	8.23	5.18	1.14	up	nch	yes	yes	yes
NM_001033319	liprin-alpha-1 isoform B	Ppfia1	8.35	4.34	1.13	up	nch	yes	yes	yes
NM_001001979	multiple epidermal growth factor-like domains	Megf10	5.93	7.08	1.07	up	nch	yes	yes	no
NM_033565	AF4/FMR2 family member 4	Aff4	8.78	5.49	1.14	up	nch	yes	no	no
NM_011250	retinoblastoma-like protein 2	Rbl2	7.67	2.90	1.14	up	nch	yes	no	no
NM_009283	signal transducer and activator of transcription	Stat1	7.91	3.09	-1.17	up	nch	yes	no	yes
NM_001164767	roundabout homolog 3	Robo3	5.54	8.02	-1.11	up	nch	yes	no	yes
NM_008880	phospholipid scramblase 2	Plscr2	4.24	11.55	1.15	up	nch	yes	yes	no
NM_175750	plexin-A4 precursor	Plxna4	6.23	20.24	-1.04	up	nch	yes	yes	yes
NM_013571	kinase suppressor of Ras 1	Ksr1	8.37	2.10	1.15	up	nch	yes	yes	yes
NM_080448	SLIT-ROBO Rho GTPase-activating protein 3	Srgap3	6.88	5.39	-2.26	up	down	yes	no	no
NM_011104	protein kinase C epsilon type	Prkce	7.25	2.92	-2.24	up	down	yes	no	no
NM_009640	angiopoietin-1 precursor	Angpt1	7.28	6.58	-6.56	up	down	yes	no	no
NM_053195	sodium/potassium/calcium exchanger 3	Slc24a3	7.59	6.61	-3.11	up	down	yes	no	yes
NM_133829	major facilitator superfamily domain-containing	Mfsd6	7.17	6.19	-3.30	up	down	yes	no	yes
NM_010170	proteinase-activated receptor 3 precursor	F2rl2	8.37	14.98	-3.43	up	down	yes	yes	no
NM_031257	pleckstrin homology domain-containing family A	Plekha2	8.78	3.53	-4.95	up	down	yes	no	no
NM_001177648	formin-binding protein 1 isoform c	Fnbp1	9.31	2.24	-4.07	up	down	yes	yes	no
NM_153408	E3 ubiquitin-protein ligase NEURL3	Neurl3	7.75	2.44	-4.12	up	down	yes	yes	yes
NM_028238	ras-related protein Rab-38	Rab38	8.22	2.38	-3.57	up	down	yes	yes	yes
NM_183355	pre-B-cell leukemia transcription factor 1	Pbx1	7.92	5.13	-2.55	up	down	yes	yes	yes
NM_175836	spectrin beta chain, brain 1 isoform 1	Spnb2	9.33	3.00	-3.43	up	down	yes	yes	yes
NM_026910	traf2 and NCK-interacting protein kinase isoform	Tnik	7.64	7.79	-2.57	up	down	yes	yes	yes
NM_011932	dual adapter for phosphotyrosine and…	Dapp1	9.83	2.63	-3.23	up	down	yes	yes	yes

Accession No	Description	Gene symbol	Expression in HSPC	Expression change in MEG, fold	Expression change in ERY, fold	Response in MEG	Response in ERY	Bound by heptad in HPC-7	Bound by heptad in HPC-7 and GATA1 in MEG at overlapping site	Bound by heptad in HPC-7 and TAL1 in MEG at overlapping site
**NM_010575**	**integrin alpha-IIb**	**Itga2b**	**8.99**	**13.15**	**-2.44**	**up**	**down**	**yes**	**yes**	**yes**
NM_009707	rho GTPase-activating protein 6 isoform a	Arhgap6	7.22	8.43	-2.40	up	down	yes	yes	yes
NM_023635	ras-related protein Rab-27A	Rab27a	8.35	2.95	-5.39	up	down	yes	yes	yes
NM_001024617	type II inositol-3,4-bisphosphate 4-phosphatase	Inpp4b	6.61	8.64	-2.22	up	down	yes	yes	yes
NM_022881	regulator of G-protein signaling 18	Rgs18	8.87	10.85	-7.83	up	down	yes	yes	yes
NM_022029	neurogranin	Nrgn	9.32	6.23	-3.85	up	down	yes	yes	yes
**NM_019932**	**platelet factor 4 precursor**	**Pf4**	**9.92**	**11.66**	**-3.32**	**up**	**down**	**yes**	**yes**	**yes**
NM_016745	sarcoplasmic/endoplasmic reticulum calcium	Atp2a3	10.07	3.90	-16.49	up	down	yes	yes	yes
NM_028133	egl nine homolog 3	Egln3	9.77	3.14	-18.63	up	down	yes	no	yes
NM_011303	short-chain dehydrogenase/reductase 3 isoform 1	Dhrs3	9.32	4.04	-3.65	up	down	yes	yes	yes
NM_027763	trem-like transcript 1 protein	Treml1	8.75	10.02	-2.16	up	down	yes	no	no
NM_021278	thymosin beta-4	Tmsb4x	11.10	2.79	-2.58	up	down	yes	no	no
NM_018729	natural killer cell receptor 2B4 precursor	Cd244	7.27	2.25	-2.33	up	down	yes	no	no
NM_015811	regulator of G-protein signaling 1	Rgs1	6.53	4.93	-3.09	up	down	yes	no	no
NM_009794	calpain-2 catalytic subunit	Capn2	7.93	6.70	-2.56	up	down	yes	yes	yes
NM_008851	membrane-associated phosphatidylinositol	Pitpnm1	7.70	3.90	-2.28	up	down	yes	yes	yes
NM_008348	interleukin-10 receptor subunit alpha precursor	Il10ra	7.83	3.57	-4.28	up	down	yes	yes	yes
NM_007796	protein CTLA-2-alpha isoform a	Ctla2a	6.66	11.50	-4.28	up	down	yes	yes	yes
NM_010819	C-type lectin domain family 4 member D isoform	Clec4d	6.16	3.84	-2.28	up	down	yes	no	no
NM_010696	lymphocyte cytosolic protein 2	Lcp2	9.76	3.29	-2.95	up	down	yes	no	yes
NM_008869	cytosolic phospholipase A2	Pla2g4a	8.51	5.50	-3.20	up	down	yes	no	no
**NM_007657**	**CD9 antigen**	**Cd9**	**8.32**	**12.01**	**-2.92**	**up**	**down**	**yes**	**no**	**no**
NM_001113460	tyrosine-protein kinase Tec isoform a	Tec	7.54	8.25	-4.19	up	down	yes	no	no
NM_198411	inverted formin-2	Inf2	7.93	4.36	-2.37	up	down	yes	yes	yes

Accession No	Description	Gene symbol	Expression in HSPC	Expression change in MEG, fold	Expression change in ERY, fold	Response in MEG	Response in ERY	Bound by heptad in HPC-7	Bound by heptad in HPC-7 and GATA1 in MEG at overlapping site	Bound by heptad in HPC-7 and TAL1 in MEG at overlapping site
NM_177855	mediator of RNA polymerase II transcription	Med12l	7.36	9.16	-2.12	up	down	yes	yes	yes
NM_172468	sorting nexin-30	Snx30	7.64	2.20	-2.22	up	down	yes	yes	yes
NM_019549	pleckstrin	Plek	9.23	12.78	-3.53	up	down	yes	yes	yes
NM_011101	protein kinase C alpha type	Prkca	7.28	15.34	-2.23	up	down	yes	yes	yes
NM_010789	homeobox protein Meis1 isoform A	Meis1	8.97	3.49	-6.33	up	down	yes	yes	yes
NM_008139	guanine nucleotide-binding protein G(q) subunit	Gnaq	8.48	5.30	-2.87	up	down	yes	yes	yes
NM_001033399	glucose-fructose oxidoreductase	Gfod1	7.10	3.14	-2.04	up	down	yes	yes	yes
NM_001081298	latrophilin-2	Lphn2	6.95	4.09	1.84	up		yes	no	yes
NM_001001491	tropomyosin alpha-4 chain	Tpm4	9.99	6.54	-1.78	up		yes	no	yes
NM_175437	gamma-secretase-activating protein	Pion	5.98	9.04	-1.36	up		yes	no	no
NM_025706	TBC1 domain family member 15	Tbc1d15	8.14	3.53	2.52	up		yes	no	no
NM_172647	junctional adhesion molecule A precursor	F11r	6.33	20.26	1.23	up		yes	no	yes
NM_146145	tyrosine-protein kinase JAK1	Jak1	9.49	3.09	2.17	up		yes	no	no
NM_133885	oxysterol-binding protein-related protein 9	Osbpl9	8.86	3.21	1.85	up		yes	no	yes
NM_009124	ataxin-1	Atxn1	7.52	3.59	-1.85	up		yes	no	no
NM_033563	Krueppel-like factor 7	Klf7	6.74	10.26	1.28	up		yes	no	no
NM_013489	SLAM family member 5 precursor	Cd84	6.35	8.92	-1.46	up		yes	no	no
NM_011866	cAMP and cAMP-inhibited cGMP 3',5'-cyclic	Pde10a	6.37	8.18	-1.39	up		yes	no	no
NM_011602	talin-1	Tln1	10.93	2.11	-1.32	up		yes	no	no
NM_011416	probable global transcription activator SNF2L2	Smarca2	7.09	3.94	-2.04	up		yes	no	no
NM_010879	non-catalytic region of tyrosine kinase adaptor	Nck2	8.60	5.89	1.81	up		yes	yes	yes
NM_011203	tyrosine-protein phosphatase non-receptor type	Ptpn12	8.28	5.99	1.81	up		yes	no	yes
NM_011145	peroxisome proliferator-activated receptor	Ppard	8.63	3.37	1.95	up		yes	no	no
NR_000002	small nucleolar RNA, C/D box 32A	Snord32a	9.96	2.04	1.85	up		yes	yes	yes
NM_172753	chondroitin sulfate	Csgalnact1	6.14	3.86	-1.69	up		yes	yes	yes

Accession No	Description	Gene symbol	Expression in HSPC	Expression change in MEG, fold	Expression change in ERY, fold	Response in MEG	Response in ERY	Bound by heptad in HPC-7	Bound by heptad in HPC-7 and GATA1 in MEG at overlapping site	Bound by heptad in HPC-7 and TAL1 in MEG at overlapping site
NM_010266	guanine deaminase	Gda	6.05	12.11	-1.67	up		yes	no	no
NM_008913	serine/threonine-protein phosphatase 2B	Ppp3ca	8.49	2.91	-1.52	up		yes	no	no
NM_134133	putative small membrane protein NID67	2010002N04Rik	9.43	5.88	1.22	up		yes	yes	yes
NM_029653	death-associated protein kinase 1	Dapk1	8.14	2.63	-1.24	up		yes	yes	yes
NM_176860	ubiquitin-associated and SH3 domain-containing	Ubash3b	7.48	13.79	-1.89	up		yes	yes	yes
NM_175353	exocyst complex component 6	Exoc6	8.30	5.00	2.90	up		yes	yes	yes
NM_011365	intersectin-2 isoform 2	Itsn2	7.76	3.30	1.79	up		yes	yes	yes
NM_133732	ester hydrolase C11orf54 homolog	4931406C07Rik	7.98	2.67	-1.97	up		yes	yes	yes
NM_001001488	probable phospholipid-transporting ATPase IC	Atp8b1	5.19	13.54	1.44	up		yes	yes	yes
NM_028643	EF-hand domain-containing family member A1	Efha1	8.45	2.56	1.74	up		yes	yes	yes
**NM_023785**	**platelet basic protein**	**Ppbp**	**10.63**	**7.25**	**-2.12**	**up**		**yes**	**yes**	**yes**
NM_019919	latent-transforming growth factor beta-binding	Ltbp1	7.25	14.75	-1.60	up		yes	yes	yes
NM_018811	abhydrolase domain-containing protein 2	Abhd2	9.10	2.25	1.58	up		yes	yes	no
NM_011682	utrophin	Utrn	7.22	4.27	-1.86	up		yes	yes	yes
NM_011347	P-selectin	**Selp**	6.20	29.49	-1.69	up		yes	yes	yes
NM_011308	nuclear receptor corepressor 1	Ncor1	8.98	4.34	2.48	up		yes	yes	yes
**NM_010326**	**platelet glycoprotein Ib alpha chain precursor**	**Gp1ba**	**6.56**	**17.26**	**-1.69**	**up**		**yes**	**yes**	**yes**
NM_001083810	proline-rich protein 5-like	Prr5l	5.26	14.02	-1.42	up		yes	yes	yes
NM_001009935	thioredoxin-interacting protein isoform 1	Txnip	10.50	2.11	-1.66	up		yes	no	yes
NM_198013	CUE domain-containing protein 1 isoform 1	Cuedc1	6.72	11.19	-1.59	up		yes	yes	yes
NM_016780	integrin beta-3 precursor	Itgb3	8.48	22.38	-2.39	up		yes	yes	yes
NM_001082553	ras-related protein Rab-27B	Rab27b	6.50	41.77	-1.43	up		yes	yes	yes
NM_001033259	coiled-coil domain-containing protein 109A	Ccdc109a	8.49	4.21	-1.37	up		yes	yes	yes

Supplementary Table 2. Developmental priming of ERY genes by the GATA2/LYL1/ TAL1/FLI1/ERG/RUNX1/LMO2 transcription factor heptad. The table shows developmentally induced ERY genes that are occupied by the heptad in HPC-7 cells (Wilson et al, 2010) and by GATA1 in maturing ERY cells, respectively, at overlapping OSs. The directionality of the developmental response in the alternate lineage is also shown along with the fold change of gene expression. Blank values indicate indeterminable response (fold change between 1.2 and 2, or FDR > 0.05). Common MEG-ERY primed genes are *italicized*.

Accession No	Description	Gene symbol	Expression in HSPC	Expression change in MEG, fold	Expression change in ERY, fold	Response in MEG	Response in ERY	Bound by heptad in HPC-7	Bound by heptad in HPC-7 and GATA1 in MEG at overlapping site	Bound by heptad in HPC-7 and TAL1 in MEG at overlapping site
NM_175367	*stonin-2*	*Ston2*	7.86	8.49	3.66	up	up	yes	no	no
NM_018784	*type 2 lactosamine alpha-2,3-sialyltransferase*	*St3gal6*	7.13	8.39	6.24	up	up	yes	no	no
NM_010433	*homeodomain-interacting protein kinase 2 isoform*	*Hipk2*	9.36	3.66	6.74	up	up	yes	no	no
NM_133167	*beta-parvin*	*Parvb*	7.86	20.29	3.75	up	up	yes	no	no
NM_010587	*intersectin-1 isoform 1*	*Itsn1*	6.42	3.73	7.12	up	up	yes	no	no
NM_138953	*RNA polymerase II elongation factor ELL2*	*Ell2*	6.87	6.52	28.53	up	up	yes	no	no
NM_028759	*DDB1- and CUL4-associated factor 6*	*Dcaf6*	7.28	2.91	5.97	up	up	yes	no	no
NM_013862	*rab GTPase-activating protein 1-like isoform a*	*Rabgap1l*	6.52	6.33	5.93	up	up	yes	no	no
NM_010026	*arf-GAP with SH3 domain, ANK repeat and PH*	*Asap1*	8.77	4.49	4.68	up	up	yes	no	no
NM_001081232	*hypothetical protein LOC320661*	*D5Ertd579e*	5.55	4.52	12.84	up	up	yes	no	no
NM_001081308	*serine/threonine-protein kinase TAO3*	*Taok3*	8.29	3.91	2.43	up	up	yes	no	no
NM_026148	*LIM and senescent cell antigen-like-containing*	*Lims1*	8.49	9.02	2.87	up	up	yes	no	no
NM_023605	*F-box only protein 9 isoform 1*	*Fbxo9*	7.63	3.15	5.66	up	up	yes	no	no
NM_172473	*E3 ubiquitin-protein ligase HACE1*	*Hace1*	7.09	3.13	10.74	up	up	yes	yes	yes
NM_145541	*ras-related protein Rap-1A precursor*	*Rap1a*	8.89	8.22	4.93	up	up	yes	yes	yes
NM_026735	*mps one binder kinase activator-like 1A*	*Mobkl1a*	7.84	7.85	3.76	up	up	yes	yes	no
NM_026921	*iron-sulfur cluster assembly 1 homolog,*	*Isca1*	8.11	3.07	7.84	up	up	yes	yes	yes

Accession No	Description	Gene symbol	Expression in HSPC	Expression change in MEG, fold	Expression change in ERY, fold	Response in MEG	Response in ERY	Bound by heptad in HPC-7	Bound by heptad in HPC-7 and GATA1 in MEG at overlapping site	Bound by heptad in HPC-7 and TAL1 in MEG at overlapping site
NM_146025	*sterile alpha motif domain-containing protein 14*	*Samd14*	8.49	4.67	5.12	up	up	yes	yes	no
NM_001164557	*PDZK1-interacting protein 1 isoform 1*	*Pdzk1ip1*	7.27	4.05	4.38	up	up	yes	yes	yes
NM_153538	*terminal uridylyltransferase 7*	*Zcchc6*	7.87	3.23	8.58	up	up	yes	yes	yes
NM_145486	*E3 ubiquitin-protein ligase MARCH2*	*40604*	9.18	3.27	6.85	up	up	yes	yes	yes
NM_028011	*TOM1-like protein 1*	*Tom1l1*	7.69	6.55	7.48	up	up	yes	yes	yes
NM_026004	*cytosolic 5'-nucleotidase 3*	*Nt5c3*	7.03	9.18	11.47	up	up	yes	yes	no
NM_001080977	*round spermatid basic protein 1-like*	*Rsbn1l*	7.65	7.35	3.84	up	up	yes	yes	yes
NM_021461	*MAP kinase-interacting serine/threonine-protein*	*Mknk1*	8.99	5.74	2.31	up	up	yes	yes	yes
NM_016920	*V-type proton ATPase 116 kDa subunit a isoform*	*Atp6v0a1*	8.60	6.08	7.31	up	up	yes	yes	yes
NM_011158	*cAMP-dependent protein kinase type II-beta*	*Prkar2b*	8.66	12.05	5.61	up	up	yes	yes	no
NM_009177	*CMP-N-acetylneuraminate-beta-galactosamide-*	*St3gal1*	9.68	2.77	2.35	up	up	yes	yes	yes
NM_001168525	*phosphatidylcholine:ceramide*	*Sgms1*	8.04	3.87	3.11	up	up	yes	yes	yes
NM_173755	*ubiquitin-conjugating enzyme E2 O*	*Ube2o*	8.52	4.85	10.65	up	up	yes	yes	yes
NM_011170	*major prion protein precursor*	*Prnp*	5.58	3.00	8.21	up	up	yes	yes	yes
NM_009179	*CMP-N-acetylneuraminate-beta-galactosamide-*	*St3gal2*	7.79	2.74	3.97	up	up	yes	yes	yes
NM_008207	*histocompatibility 2, T region locus 24*	*H2-T24*	6.79	6.76	7.21	up	up	yes	yes	yes
NM_177857	*DENN domain-containing protein 2C*	*Dennd2c*	6.27	12.94	14.80	up	up	yes	yes	yes
NM_023738	*ubiquitin-like modifier-activating enzyme 7*	*Uba7*	7.63	4.14	2.92	up	up	yes	yes	yes
NM_001033550	*leucine-rich repeat-containing protein 8B*	*Lrrc8b*	9.15	3.42	3.47	up	up	yes	yes	yes
NM_001033488	*major facilitator superfamily domain-containing*	*Mfsd2b*	9.27	4.22	3.08	up	up	yes	yes	yes
NM_146073	probable palmitoyltransferase ZDHHC14	Zdhhc14	7.15	-1.13	7.18	nch	up	yes	no	no
NM_173038	tubulin-specific chaperone cofactor E-like	Tbcel	8.27	1.17	5.26	nch	up	yes	yes	no
NM_010515	cation-independent mannose-6-phosphate receptor	Igf2r	7.55	1.10	2.88	nch	up	yes	no	no
NM_023440	transmembrane protein 86B	Tmem86b	8.09	-1.04	4.32	nch	up	yes	yes	yes
NM_018872	transmembrane protein 131	Tmem131	9.11	1.19	4.15	nch	up	yes	yes	yes

Accession No	Description	Gene symbol	Expression in HSPC	Expression change in MEG, fold	Expression change in ERY, fold	Response in MEG	Response in ERY	Bound by heptad in HPC-7	Bound by heptad in HPC-7 and GATA1 in MEG at overlapping site	Bound by heptad in HPC-7 and TAL1 in MEG at overlapping site
NM_145508	dual specificity	Dyrk3	8.00	-1.05	3.24	nch	up	yes	yes	yes
NM_018776	cytokine receptor-like factor 3	Crlf3	9.31	-1.04	2.53	nch	up	yes	yes	yes
NM_001114332	monocarboxylate transporter 10 isoform 1	Slc16a10	8.94	-1.08	6.21	nch	up	yes	yes	yes
NM_001029856	ATPase family AAA domain-containing protein 5	Atad5	6.93	-1.06	5.73	nch	up	yes	yes	yes
NM_013711	thioredoxin reductase 2, mitochondrial	Txnrd2	8.31	-2.52	5.02	down	up	yes	no	no
NM_033569	metal transporter CNNM2 isoform a	Cnnm2	7.76	-2.02	2.39	down	up	yes	no	no
NM_008256	hydroxymethylglutaryl-CoA synthase,	Hmgcs2	7.66	-3.26	2.41	down	up	yes	no	no
NM_172543	RIKEN cDNA 5730593F17	Fam117a	9.77	-3.26	2.69	down	up	yes	yes	yes
NM_001085409	metalloreductase STEAP3	Steap3	8.91	-2.92	4.26	down	up	yes	yes	no
NM_172475	FERM domain-containing protein 4A isoform 1	Frmd4a	7.36	1.68	3.86		up	yes	no	no
NM_008845	phosphatidylinositol-5-phosphate 4-kinase type-2	Pip4k2a	9.90	2.49	2.72		up	yes	no	no
NM_010019	death-associated protein kinase 2	Dapk2	6.06	-1.26	12.75		up	yes	no	no
NM_027868	solute carrier family 41 member 3 isoform 1	Slc41a3	9.44	-1.88	2.52		up	yes	no	no
NM_007964	ecotropic viral integration site 5 protein	Evi5	6.18	-1.49	11.12		up	yes	no	no
NM_007550	Bloom syndrome protein homolog isoform 1	Blm	8.33	2.18	5.10		up	yes	no	no
NM_173402	regulator of G-protein signalling 12 isoform A	Rgs12	7.71	1.43	2.29		up	yes	no	no
NM_001081636	G1/S-specific cyclin-D3	Ccnd3	10.30	1.94	2.08		up	yes	no	no
NM_001001184	coiled-coil domain-containing protein 111	Ccdc111	7.36	2.18	2.84		up	yes	yes	yes
NM_028419	glutaredoxin-related protein 5, mitochondrial	Glrx5	10.82	-1.31	3.11		up	yes	yes	yes
NM_013454	ATP-binding cassette sub-family A member 1	Abca1	7.68	1.96	2.13		up	yes	no	no
NM_177391	hypothetical protein LOC338368	Fam109b	7.45	-1.79	3.15		up	yes	yes	yes
NM_033623	DCN1-like protein 1	Dcun1d1	7.80	3.10	5.05		up	yes	yes	yes
NM_029081	hypothetical protein LOC74741 isoform 1	5730419I09Rik	8.51	1.34	3.28		up	yes	yes	yes
NM_028932	ELL-associated factor 1	Eaf1	8.71	-1.29	2.55		up	yes	yes	yes
NM_177733	transcription factor E2F2	E2f2	7.52	-1.26	16.07		up	yes	yes	yes

Accession No	Description	Gene symbol	Expression in HSPC	Expression change in MEG, fold	Expression change in ERY, fold	Response in MEG	Response in ERY	Bound by heptad in HPC-7	Bound by heptad in HPC-7 and GATA1 in MEG at overlapping site	Bound by heptad in HPC-7 and TAL1 in MEG at overlapping site
NM_134021	pyridoxine-5'-phosphate oxidase	Pnpo	9.47	-2.12	5.18		up	yes	yes	yes
NM_019744	nuclear receptor coactivator 4	Ncoa4	9.94	2.15	5.06		up	yes	yes	yes
NM_009764	breast cancer type 1 susceptibility protein	Brca1	7.37	-1.49	5.56		up	yes	yes	yes
NM_027872	solute carrier family 46 member 3 precursor	Slc46a3	7.43	1.46	4.06		up	yes	yes	yes
NM_007970	histone-lysine N-methyltransferase EZH1	Ezh1	8.26	1.88	2.10		up	yes	yes	yes
NM_001033988	nuclear receptor coactivator 4	Ncoa4	9.86	2.10	4.87		up	yes	yes	yes
NM_020332	progressive ankylosis protein	Ank	8.03	2.01	4.50		up	yes	yes	yes
NM_010576	integrin alpha-4	Itga4	9.81	-1.74	3.43		up	yes	yes	yes
NM_029332	A kinase (PRKA) anchor protein 13	Akap13	8.13	1.43	2.23		up	yes	yes	yes
NM_001033301	FH2 domain-containing protein 1	Fhdc1	5.13	1.14	70.24		up	yes	yes	no
NM_009569	zinc finger protein ZFPM1	Zfpm1	10.39	1.57	2.27		up	yes	yes	yes
NM_008654	protein phosphatase 1 regulatory subunit 15A	Ppp1r15a	8.14	1.81	6.90		up	yes	yes	yes
